# Review: Detection of Cancer Biomarkers from a Clinical Perspective

**DOI:** 10.3390/ijms262311745

**Published:** 2025-12-04

**Authors:** Xeniya Terzapulo, Aigerim Dyussupova, Aisha Ilyas, Aigerim Boranova, Yegor Shevchenko, Saule Mergenbayeva, Aiym Kassenova, Olena Filchakova, Abduzhappar Gaipov, Rostislav Bukasov

**Affiliations:** 1 Chemistry Department, School of Sciences and Humanities, Nazarbayev University, Kabanbay Batyr Ave. 53, Astana 010000, Kazakhstan; xeniya.terzapulo@nu.edu.kz (X.T.);; 2 Biology Department, School of Sciences and Humanities, Nazarbayev University, Kabanbay Batyr Ave. 53, Astana 010000, Kazakhstan; 3 Department of Medicine, Nazarbayev University School of Medicine, Astana 010000, Kazakhstan

**Keywords:** cancer biomarkers, AUC, clinical diagnostics, SERS, FTIR, electoanalytical methods

## Abstract

Cancer is the disease found to be the reason for the largest portion of deaths in the world annually and these mortality values are expected to increase in the future. Early detection of cancer biomarkers may help save millions of lives, particularly by implementing non-invasive and economical detection methods. In this review, we tabulated and quantitatively compared the data collected in 173 rows from 124 publications, which describe the clinical application of various methods in detection of cancer biomarkers. Those methods include mass spectrometry (MS), immunoassays (IAs), enzyme-linked immunosorbent assay (ELISA), polymerase chain reaction (PCR), surface-enhanced Raman spectroscopy (SERS), and Fourier-transform infrared spectroscopy (FTIR). We found that direct methods may have an advantage over indirect methods. Direct SERS reported in clinical applications can also achieve a higher area under the curve, higher sensitivity, and specificity than those parameters for ELISA, PCR, MS, and FTIR applications. Based on the average area under the curve (AUC) values reported in the last 6–7 years for each method, the performance of the analytical methods for the clinical cancer detection increases from IAs (0.76), ELISA (0.83), MS (0.87), and PCR (0.89) to FTIR (0.95) and SERS (0.97).

## 1. Introduction

Cancer is a disease characterized by uncontrollable growth of the cells with possible spread to other parts of the body and other tissues [1]. The first description of this disease was written in approximately 3000 BC in the Edwin Smith Papyrus, where the first evidences of breast cancer were shown [2]. Cancer is not evenly distributed across the countries, but it is predicted that the worldwide cancer incidence is going to reach 25 million by 2040 [3]. There are numerous types of this disease known nowadays and according to the Cancer Statistics in the US for 2023, the breast and prostate cancers were expected to be the most widespread among women and men, respectively [4]. The highest death rate was expected among patients of both sexes with lung and bronchus cancer, which is also found to be in second place when analyzing estimated incidence cases [4].

Cancer-related disability-adjusted life years (DALYs) among individuals aged ≥ 65 years increased by 95.14% between 1990 (52.25 million) and 2019 (101.96 million) [5]. Cancer-related DALYs which attributed to population aging followed a bell-shaped pattern when stratified by SDI, meaning they peaked in middle-SDI countries. Cancer-related DALYs which attributed to aging increased in 171 countries/territories and decreased in 33. Since some predictions suggest that older adults will comprise 61% of the population in 155 countries by 2100, the cancer impact on global health and wellbeing may become significantly worse than it is now [5].

The cancer is usually detected by analyzing the biomarkers, such as cancer cells, nucleic acids, proteins, enzymes, and other small chemical products [6]. The detection of the cancer biomarkers plays an essential role in the decrease in the death rate of cancer, since the survival rate of patients with cancer that was detected at low concentrations is expected to be higher than that of people with a later stage of disease detected. Saadatmand et al. illustrated in their research the importance of early detection by presenting the survival rate of patients with breast cancer detected in the different stages of disease development [7]. For tumor stage T1a, where the size of the tumor is ≤1 cm, the relative survival rate was equal to 100%, whereas for T4 stage which describes any size of tumor with direct extension to the chest wall and/or skin, the rate was equal to 59% [7]. The authors also suppose that the survival rate is found to be higher since only local surgery measures are needed to be implemented if cancer was detected at early stages in comparison with chemotherapy and other measures that are usually taken if cancer was detected in later stages [7].

Multiplexing is also considered to be beneficial to the techniques that are usually used for the cancer detection. The possibility of the detection of several cancer biomarkers is usually used to facilitate the analysis and detect the disease at early stages [8]. For example, Chen et al. developed reversed phase liquid chromatography coupled with a quadrupole time-of-flight mass spectrometry (RPLC-QTOF/MS) method for detecting six metabolic biomarkers of laryngeal cancer in urine. Research outcomes presented that sensitivity, specificity of the ROC curve and area under the curve (AUC) were 95%, 97%, and 0.97, respectively [9]. Furthermore, multiplexing provides the opportunity to perform analyses in more cost-effective and rapid ways without negative impact on the sensitivity and selectivity of the biosensors due to the improved repeatability [10].

Similarly to multiplexing benefits, the sensitivity and accuracy for the detection of cancer biomarkers can be improved by integrating computational tools such as machine learning (ML) for early clinical diagnosis with enhanced accuracy, sensitivity, and specificity parameters. Machine learning (ML) includes the type of predictive models that are actively used in the cancer detection procedures [11]. Such models can be used to predict the presence or absence of a tumor, as well as describe their malignancy [11]. There are numerous databases that are actively used to detect, describe, and monitor different types of cancers such as breast, brain, lung, liver, and skin cancer as well as leukemia [11,12].

There are multiples reviews on cancer detection but most of them are focused on one analytical technique or on a certain type of biological fluid used for the biomarker detection. There are such reviews that were concerned with the detection of cancer biomarkers using such techniques as mass spectrometry (MS) [13], polymerase chain reaction (PCR) [14], ELISA [15], immunoassays (IAs) [16], surface-enhanced Raman scattering (SERS) [17], Fourier-transform infrared spectroscopy (FTIR) [18] in biological fluids such as blood [19], urine [20], and saliva [21]. Other advanced techniques, such as Atomic Force Microscopy (AFM), can be used for cancer biomarker detection via morphological, mechanical, and chemical characterization of the molecular processes [22]. Next-Generation Sequencing (NGS) is also found to be an alternative nanoscale imaging tool, which is a very useful technique for the precise and sensitive detection of cancer biomarkers at early stages and plays a significant role in precision oncological therapy and disease monitoring [23].

There is just one comprehensive review, that we know of, about the detection of cancer biomarkers from an analytical perspective, where major analytical and bioanalytical methods are compared in terms of LOD and other analytical parameters (concentration range, RSD %, etc.) reported for detection of cancer biomarkers [24]. However, there are no comprehensive reviews that compare the abovementioned detection methods from their clinical application perspectives. In addition, most reviews are based on the description of analytical parameters such as limit of detection (LOD), linear range, and, sometimes, the reproducibility of the results.

This review will focus on the clinical applications of various analytical techniques such as MS, PCR, ELISA, IAs, SERS, and FTIR. Reported results were used to create the broad picture of the implementation of the abovementioned methods from a clinical perspective.

## 2. Clinical Detection Using Different Methods

### 2.1. Detection with Mass Spectrometry

Initially this technique was used to measure atomic masses and show isotopes in the early 20th century and it was developed by a few dedicated proponents [25]. By the 1940s, it was adopted into the petroleum industry to analyze small hydrocarbon abundances in process streams [25]. In 2002, a study claimed that protein fingerprinting in serum could diagnose ovarian cancer with a sensitivity of 100% and specificity of 95% [26]. However, the results were found to be artifacts and this beginning led to challenging searches for biomarker detections based on the mass spectrometry of the clinical proteomics [26].

Mass spectrometry, which is frequently combined with GC (Gas Chromatography) or HPLC (High-Performance Liquid Chromatography) as separation methods, operates by the separation and quantification of ions according to their mass-to-charge ratio, typically after analyte molecules are ionized. The degree of fragmentation of analyte into ions (and therefore complexity of the mass spectrum) can be controlled by selecting a harder (electron impact ionization), softer (chemical ionization), or much softer method of ionization like MALDI (matrix-assisted laser desorption ionization), which provides very low fragmentation and therefore is highly beneficial for the analysis of large molecules, for instance antibodies.

Mass spectrometry is a powerful and versatile analytical technique, which has been labeled as the queen of quantitative chemical analysis; it combines high sensitivity, sometimes with LOD (limit of detection) near ppt level (10^−12^ g analyte/1 g sample or about 1 pg of analyte in 1 mL of solution), and a very broad range of analytes that MS can detect from elements like metals to large macromolecules with molecular weight approaching 1000 kDa. MS is frequently performed with time-of-flight mass analyzers. The resolution of MS (m/Δm) can vary by about three orders of magnitude from quadrupole to Fourier-transform ion cyclotron resonance (FTICR) MS, which can achieve impressive resolution of several millions. The relative disadvantage of MS is its high cost, which among several factors is driven most of all by a requirement to maintain a high operational vacuum (10^−8^ to 10^−12^ bars) in mass analyzers. There are numerous review papers that describe particular methods of MS and MS operation in principle as well as applications of MS in metabolomics [27,28].

The MS and machine learning methods can be merged to differentiate between clinical groups, to make precise predictions, and develop appropriate strategies for further therapy [29]. For example, Taguchi et al. used the K-nearest neighbor (KNN) algorithm method to process matrix-assisted laser desorption ionization (MALDI) mass spectra. They identified a group of patients among two independent validation cohorts of 67 and 96 patients that have statistically significant survival rates after gefitinib treatment against non-small cell lung cancer (NSCLC). The concordance between two institutions where the classifications based on spectra were acquired was equal to 97.1%, demonstrating a relatively good classification ability of the method [29].

The clinical papers published about cancer biomarkers detection by MS in the last decade are summarized in Table 1. The calculated average of sensitivity, specificity, and accuracy for reported works were 86.5%, 86.35%, and 89.12%, respectively. The median of sensitivity, specificity, and accuracy were 88.2%, 86%, and 90.2%, respectively. The diagnostic sensitivity of 95%, specificity of 97%, and area under the curve (AUC) of 0.97 were obtained by recognizing metabolites as potential biomarkers of laryngeal cancer in human urine of 66 patients [9]. As another example, for the identification of colorectal cancer, the sensitivity and specificity of the derivatization liquid chromatography–mass spectrometry technique in the paper by Bian et al. [30] were notably better (sensitivity: 96.9% vs. 92.6%, specificity: 94.4% vs. 91.7%) than the GC-MS method [31], where prostate cancer biomarkers were studied.

**Figure 1:**
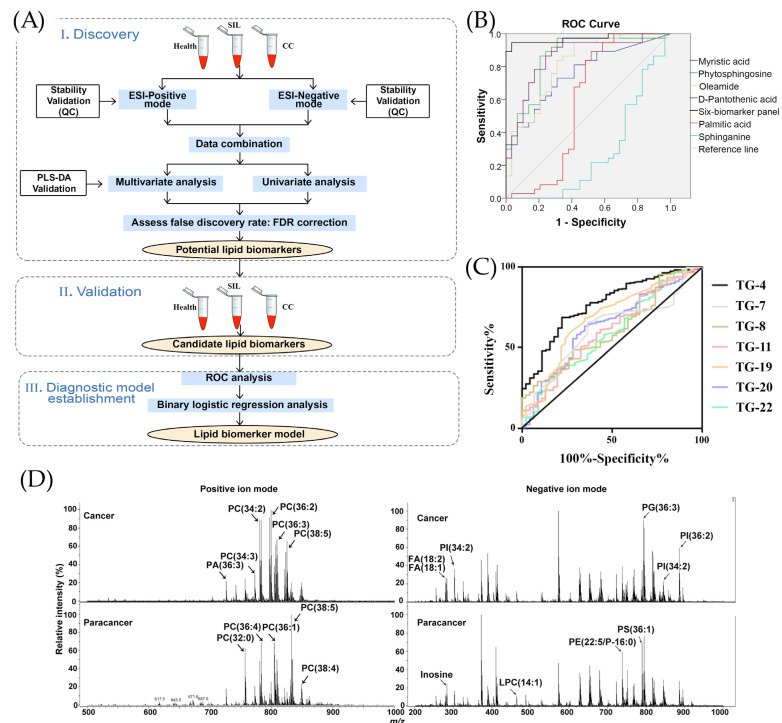
The performance of MS techniques for the detection of different cancer types. (**A**) The schematic representation of the lipidomic research for cervical cancer (CC) [42], adopted with permission from Cheng et al. [42] Copyright © 2020 Elsevier V.B. (**B**) Receiving operator characteristic (ROC) curve for identifying laryngeal cancer (LYC) patients with six metabolites (myristic acid, phytosphingosine, oleamide, D-pantothenic acid, palmitic acid, sphinganine) by UPLC-QTOF/MS, adopted with permission from Chen et al. [9] Copyright © 2019 Elsevier V.B. (**C**) ROC curves for various VOCs in lung cancer detection: 3-hydroxy-2-butanone (TG-4), glycolaldehyde (TG-7), 2-pentanone (TG-8), acrolein (TG-11), nonaldehyde (TG-19), decanal (TG-20), and crotonaldehyde (TG-22) [61], reused with permission from Sani et al., *Cancers*, published by MDPI, 2023 [61]. (**D**) Illustration of mass spectra of the cancerogenic and paracancerous regions, reproduced with permission from Zou et al. [55] © 2022 Elsevier B.V.

There is a proposed method for the detection of tumor biomarkers for cervical cancer (CC) using the lipidomic analysis performed using UHPLC/Q-TOF-MS by Cheng et al. [42]. As illustrated in Figure 1A, the process contains discovery, validation, and establishment of the diagnostic model, where lipid biomarkers are analyzed by UHPLC-Q-TOF-MS after their successful extraction from serum [42]. The diagnostic performance of these biomarkers were assessed by an analysis of ROC curves, corresponding to which phosphatidylethanolamine (PE) reached an AUC value of 0.9 [42].

Chen et al. studied various types of urinary metabolites for the detection of laryngeal cancer (LYC) using the metabolomics and applied it in clinical tests for LYC patients and healthy controls (HC). The base peak chromatograms were compared in MS/MS spectra and their retention times in the spectra were observed for compounds producing the signals [9]. The significant changes were observed in six metabolites that are myristic acid, phytosphingosine, oleamide, D-pantothenic acid, palmitic acid, and sphinganine; LYC was diagnosed by means of the ROC curves [9]. As shown in Figure 1B, the highest sensitivity of 97% was detected for phytosphingosine with AUC = 0.86 and specificity of 69% [9]. The parameters of the combined six metabolites showed sensitivity and specificity of 95% and 97%, respectively, and an AUC of 0.97 [9].

Similarly, Sani et al. studied the use of volatile organic compound (VOC) biomarkers for the screening of lung cancer [61]. In comparison with healthy controls, patients in early stages of lung cancer showed great differences in the following VOCs: 3-hydroxy-2-butanone (TG-4), glycolaldehyde (TG-7), 2-pentanone (TG-8), acrolein (TG-11), nonaldehyde (TG-19), decanal (TG-20), and crotonaldehyde (TG-22) [61]. According to analysis of ROC curves in Figure 1C, 3-hydroxy-2-butanone demonstrated the greatest difference with a sensitivity of 70% and specificity of 76% [61]. The combined analysis of seven compounds allowed a sensitivity and specificity of 83% and 78%, respectively [61].

Figure 1D illustrates the mass spectra of one cancerogenic region. Zou et al. reached 100% sensitivity and 100% specificity and an AUC of 1, determining and analyzing serum phosphatidylcholine (32:0) as a potential biomarker of early gastric cancer [55]. The liquid extraction–electrosonic spray ionization with Fourier-transform ion cyclotron resonance mass spectrometry imaging (LE-ESSI FTICR-MSI) method was performed to examine cancerogenic and paracancerous cells of six early gastric cancer patients to evaluate their human blood lipid levels. A similar improvement in clinical detection accuracy was reported in dual-mode chromatographic-MS methods. For instance, a dual-mode HPLC-MS/MS detected six urinary pteridines linked to breast cancer with high sensitivity (LOD 0.025–0.5 μg/L) and with spiked recoveries (81–105%) [62]. Similarly, Yie et al. (2006) proposed a dual method of RT-PCR ELISA for detection of cancer cells in the peripheral blood of breast cancer [63]. Their proposed technique detected Survivin-expressing circulating breast cancer cells in 50.7% of 67 patients, and none for the healthy control group [63]. During a 36 month follow-up period, 81.8% of Survivin-positive patients relapsed compared with 33.3% of negative patients, demonstrating a valuable method to predict the metastasis of breast cancer [63].

### 2.2. Detection with Immunoassays Including ELISA

The use of immunoassays in clinical chemistry was first started with Solomon Berson and Rosalyn S. Yalow’s development of the first immunoassay for insulin in 1959 [64]. Immunoassays, including the widely used ELISA (enzyme-linked immunosorbent assay), operate on the principle of highly specific molecular recognition between an antigen and its corresponding antibody. This specificity enables selective detection of target biomolecules even in complex biological matrices. In a typical immunoassay, antibodies bind to the analyte of interest, and this interaction is converted into a measurable signal through a reporter system such as an enzyme, fluorophore, or chemiluminescent label. ELISA, one of the most established immunoassay formats, generally relies on immobilizing either the antigen or antibody on a solid surface, followed by sequential binding steps and signal generation via an enzyme-catalyzed reaction. Depending on the assay configuration (direct, indirect, sandwich, or competitive), the sensitivity and detection range can be tuned to accommodate analytes ranging from small molecules to large proteins. Immunoassays are valued for their high specificity, relatively low cost, and suitability for high-throughput analysis. Their limits of detection often reach the picomolar to femtomolar range, particularly in signal-amplified chemiluminescent formats. A limitation of immunoassays, however, is their dependence on high-quality antibodies, which can introduce variability due to batch-to-batch differences and stability issues. The key diagnostics parameters in immunoassay-based methods, such as sensitivity and specificity, depend on the affinity between antigen and antibody, as well as on specificity of binding. Thus, low parameters could be the result of low affinity as well as non-specific binding of an antibody to other substances present in the solution. To overcome the low affinity issue, a setup with primary and secondary detection antibodies is used, where an unlabeled primary antibody recognizes the antigen, while a labeled secondary antibody binds to the primary antibody. Due to multiple secondary antibodies being bound, the amplification of the signal is achieved. To overcome the issue concerning non-specific binding, a blocking step process is carried out which removes the potential sites for the non-specific binding. Immunoassays that use fluorescence as a readout also depend on the parameters and the properties of the fluorescent dye, such as quantum yield and bleaching, which is similar to a decrease in fluorescent intensity with time and quenching. Numerous reviews describe the mechanistic details of antigen–antibody interactions, assay formats, and the broad applications of immunoassays in diagnostics, biomarker quantification, and pharmaceutical analysis [65,66].

Building on the foundations of radioimmunoassay (RIA), the ELISA method itself was developed by Engvall and Perlmann in 1971 as a non-radioactive alternative, replacing radioisotopes such as iodine-125 with enzyme labels [67]. Another team used an enzyme-based method to detect human chorionic gonadotropin in urine during that same year [67]. As of 2014, common methods for detecting cancer biomarkers included techniques such as enzyme-linked, radio, or electrophoretic immunosorbent assays (ELISA) as well as mass spectrometry-based proteomics [68].

The studies focused on the clinical performance of immunoassays and ELISA have been summarized in Table 2.

**Figure 2:**
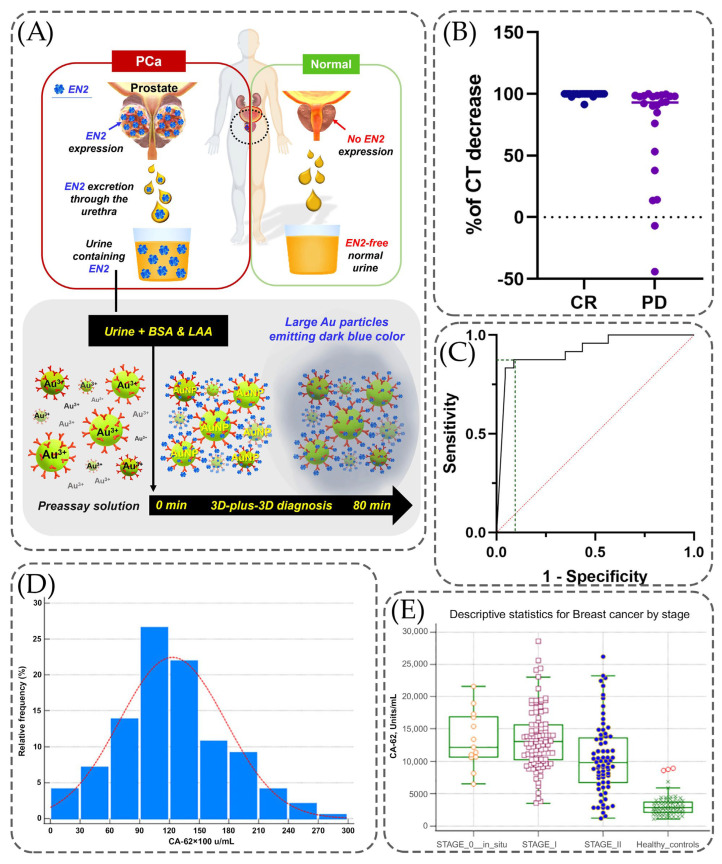
Clinical performance of the implementation of immunoassays into detection techniques. (**A**) Detection mechanism of prostate cancer-specific urinary EN2 using 3D-plus-3D immunoassay, adopted with modification from Kim et al. [91] under the terms of the Creative Commons Attribution License (CC BY). (**B**) Scatter plot presenting the calcitonin decreasing rate, comparing complete response vs. persistent disease. (**C**) ROC curve analysis considering the decreasing rate of calcitonin values, showing the levels of sensitivity and specificity, adopted with permission from Sparano et al. [89] under the terms of the Creative Commons Attribution License (CC BY). (**D**) Comparison of CA-62 levels in serum using samples of healthy controls and from all breast cancer groups. (**E**) Minimum and maximum values presented using error bars for different stages of breast cancer, adopted with modification from Sekacheva et al. [90] under the terms of the Creative Commons Attribution License (CC BY).

Figure 2 contains information regarding the cancer biomarkers detection using immunoassays. The process of detecting the urinal biomarker EN2 using a 3D-plus-3D immunoassay is shown in Figure 2A. Urine samples containing this biomarker (PCa-patient urine), ascorbic acid (LAA), as well as bovine serum albumin (BSA) are added to a preassay solution that already contains antibodies (anti-EN2 3D-IgG) and gold particles. As a result of this reaction, an immunoassay is obtained, which can then be used to detect the level of urinal EN2 in the obtained samples. Figure 2B contains a scatter plot, which presents a comparison of the decrease in calcitonin levels in the complete response and persistent disease. The decrease in the level with complete response is kept at a level equal to 100% calcitonin level with minor decreases, while for persistent disease, the level of decrease varies by as much as −50%. Despite this, most of the points shown in this graph are also at 100%, but with several points ranging from 100% to −50%. The ROC curve for the decreasing rate of calcitonin values is presented in Figure 2C. The baseline was created based on the graph presented in Figure 2B. It can be concluded that the highest sensitivity was faced along with highest specificity but there are some regions in the graph where sensitivity was higher than 80%, whereas specificity was around 50%. For example, Sparano et al. present the situation where, according to which the CT level was reduced to 95%, the sensitivity value was equal to 95.8% while the specificity resulted in a decrease to 56.5%. Figure 2D demonstrates the relative frequency of different concentrations of breast cancer biomarker CA-62 in serum samples of healthy controls and all breast cancer groups. As it has been mentioned, most often there are samples with a concentration of this biomarker equal to 9000–12,000 u/mL with a relative frequency equal to 25–26%. Next in frequency (approximately 22%) is a concentration equal to 12,000–15,000 u/mL. This suggests that among all the analyzed samples, significantly higher serum CA-62 levels are more common than lower concentrations. Figure 2E shows changes in the level of CA-62 in the blood depending on the stage of the disease. In the samples of the control group, the level of this disease, considering the minima and maxima indicated by error bars, is most often in the range from 0 to 5000 units/mL. For Stage 0 (in situ), the level of this biomarker varies on average from 10,000 to 15,000 u/mL, while reaching a maximum of approximately 22,000 u/mL and a minimum of about 6000 mL. For Stages 1 and 2, there is a trend that the level of this biomarker is higher for Stage 1, and is on average 13,000 U/mL, while for Stage 2, the average concentration is approximately 9000 U/mL. This may indicate that this biomarker is released in significant concentrations at the initial stages of carcinogenesis, when cancer cells are not yet differentiated.

**Figure 3:**
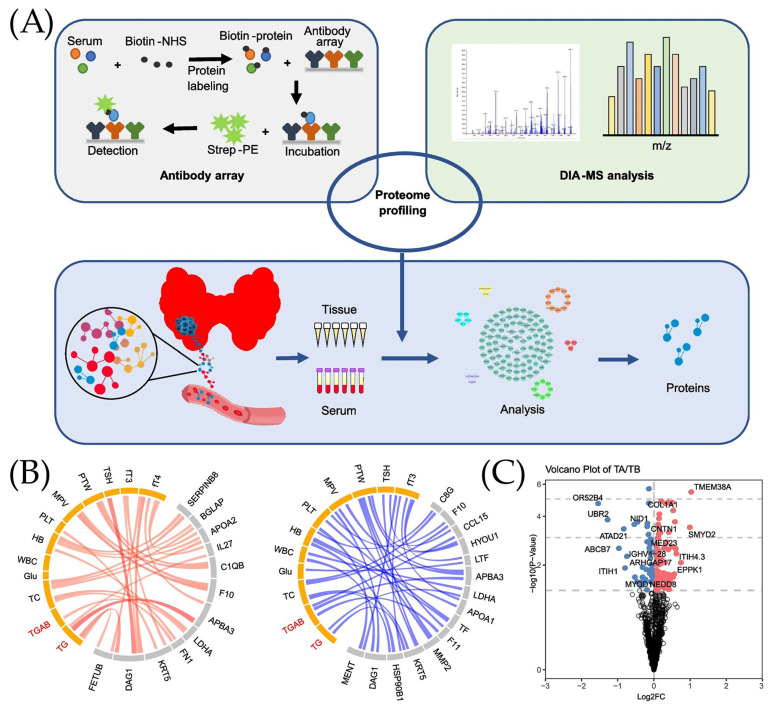
Clinical performance of ELISA technique. (**A**) Working principal of in-depth papillary thyroid cancer (PTC) serum and tissue proteomics. (**B**) Circus diagram used for presentation of positive (red) and negative (blue) correlations between serum proteome and clinical data. (**C**) Volcano plot analysis used for the identification of proteins associated with papillary thyroid cancer (PTC) in serum before and after surgery, adopted from Ye et al. [74] under the terms of the Creative Commons Attribution License (CC BY).

Figure 3 presents the papers based on the clinical performance of ELISA techniques in cancer biomarkers detection. The schematic representation of the working process of papillary thyroid cancer (PTC) serum and tissue proteomics is presented in Figure 3A. As it has been mentioned, to create the antibody array, several steps need to take place. It starts with the combination of serum and Biotin-NHS to obtain the labeled Biotin-protein. This labeled protein was later combined with an antibody array. After incubation, Strep-PE was added, making the resulting molecule suitable to be used in the detection process. Samples were taken from both serum and tissue to obtain proteins. In addition, DIA-MS analysis was also performed according to the scheme presented in Figure 3A. To differentiate the proteomic changes from the clinical data, the Circus diagram and Volcano plot were created (Figure 3B,C). The circular network plots show the relationship between various biomarkers of papillary thyroid cancer (PTC), where positive correlations are indicated in red and negative correlations in blue. It can be noted that the stronger the correlation, the thicker the line shown in the diagram, and that biomarkers are divided into categories, which are highlighted in different colors (yellow and gray) in the diagrams. Since the blue scheme is more populated, it can be concluded that there are more negative correlations in the graph than positive ones. In addition, it can also be noted that two biomarkers are highlighted in red text, which may mean that the main markers of interest (thyroglobulin (Tg) and Tg antibodies (TgAb)) have outstanding correlations with other biomarkers. A Volcano plot (Figure 3C) was used to demonstrate changes in biomarkers of two categories: TA (test after surgery of PTC patients), presented on the left, and TB (test before surgery of PTC patients), presented on the right. It can be noted that Log2FC, which means fold change, is higher for the TB category than for TA. The importance of these changes in the value of PTC biomarkers is presented using the −log10 (*p*-value) function. The points that are located on the graph above have higher expression differences between TA and TB.

### 2.3. Nucleic Acid-Based Detection of Cancer Biomarkers

Polymerase chain reaction (PCR) is a method for the amplification of nucleic acids through repeated cycles of denaturation, primer annealing, and extension using a DNA polymerase. In the 1980s, PCR was originally designed to detect the HBB gene responsible for sickle cell anemia [92]. Over the years, PCR has become a significant tool in designing biosensors with high sensitivity, as highlighted by Du and Dong [93]. Paired with colorimetry and fluorescence, the detection range of PCR extends from viral and bacterial genomes to tumor markers. The prominence of PCR increased during the COVID-19 pandemic, as reverse transcriptase PCR served as the gold-standard diagnostic tool for SARS-CoV-2, as recommended by the WHO, and new real-time and point-of-care biosensors were developed accordingly [94]. In contrast to PCR, which requires precise temperature cycling and specialized equipment, loop-mediated isothermal amplification (LAMP) is more suitable for point-of-care applications. LAMP is a one-step isothermal reaction at around 60–65 °C that provides high specificity due to the use of a set of four primers for the recognition of 6–8 target regions; it was first proposed by Notomi et al. [95] in 2000 and is now implemented in commercial diagnostic test kits [96]. Besides LAMP, other isothermal amplification techniques were proposed with the potential to be developed into low-cost point-of-care diagnostics tools. They include recombinase polymerase amplification, strand displacement amplification, rolling circle amplification, and CRISPR/Cas-based techniques. Early diagnosis of the cancer disease includes significant research to identify and validate biomarkers; one of the prominent examples to do this is immuno-PCR that detected CA 15-3 biomarker in 2014 [97]. The analytical detection of tumor DNA (ctDNA) using PCR and other nucleic acid-based approaches is also discussed in a review by D’Agata et al. in 2017 [98].

The reported application of nucleic acid-based detection methods on clinical diagnosis of various types of cancer is presented in Table 3. Polymerase chain reaction (PCR), which is the golden standard for COVID-19 virus detection [92], is also frequently used in the clinical diagnosis of tumors (28 out of 32 studies).

The clinical performance of nucleic acid-based detection of cancer biomarkers are described in Table 3, demonstrating an average sensitivity of 84.1%, specificity of 84.8%, and accuracy of 84.9%.

The DNA/RNA amplification techniques, such as quantitative and droplet digital PCR and LAMP, are often used in the clinical detection of cancer, as shown in Table 3. The median sensitivity/specificity/accuracy of the overall nucleic acid-based detection is 86%/86%/84%. The median accuracy of droplet digital PCR is slightly better than that for quantitative PCR, 83.4% and 85.5%, correspondingly. The median accuracy of the LAMP-based diagnosis is about 98.3%, while for all PCR assays it is 83.4%. The reliability of the conclusion concerning the overall diagnostic performance of LAMP-based techniques can be limited due to the low number (four studies) of reported studies in the following review.

Abnormal methylation occurring at CpG sites within promoter regions can contribute to tumor formation. The evaluation of methylation in cell-free DNA that can originate from cancer tissue is one of the methods for cancer screening. MethyLight PCR was used to study the DNA methylation of four gene promoters in plasma and tissue biopsy samples in a study by Bartak et al. [105]. Increased methylation was observed in adenoma and cancerous samples for both the plasma and tissue. Despite the low amount of cell-free DNA, the methylation pattern was determined in a two-step amplification: (1) bisulfate-specific PCR pre-amplification and (2) methylation-specific qPCR. Based on the promoter DNA methylation level, the colorectal cancer samples were discriminated from the healthy samples with 92% sensitivity and 97% specificity with an AUC of 0.978 [105].

Shinjo et al. detected pancreatic cancer using DNA methylation markers and droplet digital PCR (ddPCR) [125]. At first, five markers were selected by generating a genome-wide profile of the DNA methylation status of 37 fine-needle aspiration samples of pancreatic cancer via the Illumina Infinium HM450 Beadchip. DNA methylation in five genes in cell-free DNA could not be reproducibly detected using pyrosequencing analysis or bisulfite-based DNA methylation, as the latter is prone to DNA loss or degradation during the bisulfite treatment. Hence, the methyl-CpG binding (MBD) protein was coupled with the ddPCR method. This method allows the detection of a very limited amount of cfDNA due to the impartial amplification of DNA fragments captured by MBD by an adapter before ddPCR. Two independent assay validation studies were performed and found (1) 100% accuracy for 46 pancreatic and 6 normal samples and (2) 96% sensitivity and 90% specificity for 137 cancer patients and 10 healthy patients [125].

The electrochemistry method was combined with LAMP by Moranova et al. to detect two prostate cancer biomarkers [129]. Prostate-specific antigen (PSA) is frequently applied as a prostate cancer biomarker; however, the specificity of the assays might be limited due to the elevated levels of PSA in non-malignant prostate diseases as well. For this reason, a long non-coding RNA biomarker, prostate cancer antigen 3 (PCA3), was used as a biomarker along with PSA mRNA (that served as a control) in the following research. After the series of experiments, it was identified that the assay can be optimized using four sets of PCA3 and four sets of PSA LAMP primers. In addition, 66 °C LAMP reaction temperature and an incubation time of 75 min lead to enhanced amplification. To prevent the non-specific annealing of the primers and to increase the specificity of the LAMP reaction, a short treatment of total RNA with DNase I was added. This was required to remove all DNA traces so that PCA2 lncRNA was the only product. Amplification products were hybridized on magnetic beads and the signal outputs were detected via chronoamperometry. The stability of the prepared magnetic beads was around 3 weeks, after which a 20% drop in signal was observed. This minimally invasive assay was in 100% alignment with the clinical results for eleven prostate samples and seven healthy urine samples [129].

**Figure 4:**
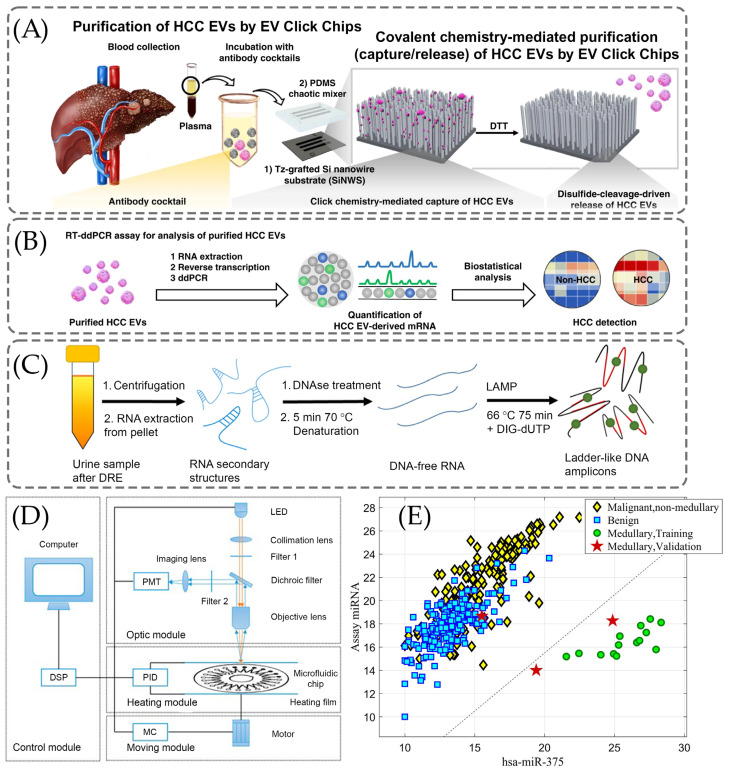
The clinical use of the PRC for diagnosis of various cancer diseases. (**A**) Mechanism of extracellular vesicle (EV) click chips that capture/release hepatocellular carcinoma (HCC) EVs from blood samples, reproduced from Sun et al. [124] under the Creative Commons Attribution 4.0 International License. (**B**) The detection of HCC by RT-ddPCR, reproduced from Sun et al. [124] under the Creative Commons Attribution 4.0 International License. (**C**) The schematic representation of the electrochemical (EC) assay-based loop-mediated isothermal amplification (LAMP) on urine samples [129], adopted with permission from Moranova et al. [129] Copyright © 2022 Elsevier V.B. (**D**) The schematic illustration of the real-time fluorescence detector with the following devices: photomultiplier (PMT), light-emitting diode (LED), proportional integral derivative (PID), moving controller (MC), and digital signal processor (DSP) [128], reused with permission from Lin et al. [128] under the Creative Commons Attribution 4.0 International License. (**E**) Results of linear discriminant analysis (LDA) in medullary carcinoma [104], adopted with permissions from Lithwick-Yanai et al. [104] under the Creative Commons Attribution 4.0 International License.

Sun et al. performed the clinical diagnosis of hepatocellular carcinoma by using a HCC extracellular vesicle purification system that consists of covalent click chemistry, multi-marker cancer antibodies, Si nanowire substrate, and microfluidic PDMS mixer [124]. EV is captured from the plasma sample by using a click chemistry reaction between tetrazine-grafted SiNWs and trans-cyclooctene-grafted HCC EVs. Afterward, the exposure to 1,4-dithiothreitol leads to the cleavage of disulfide bonds and consequently the release of EVs. (Figure 4A). This is followed by quantification of the EV-derived cancerous mRNA using reverse transcriptase dd-PCR, as displayed in Figure 4B. By applying the proposed click chemistry-based method, Sun et al. achieved the non-invasive early detection of HCC with 94% sensitivity and 89% specificity for 158 plasma samples [124].

As illustrated in Figure 4C, Moranova et al. developed an assay for the detection of the following RNA biomarkers: prostate-specific antigen (PSA) mRNA and prostate cancer antigen 3 (PCA3) lncRNA [129]. In their method, they used magnetic beads (MB) to capture RNAs selectively and the LAMP technique to amplify them [129]. The EC assay was enabled to enhance the sensitivity of the detection [129]. The treatment of isolated RNAs with DNase I was applied to eliminate any DNA traces and they were further denatured by heating to remove secondary structures of RNA [129]. DNA-free RNA structures were added to a mixture containing PSA and PCA3 primers and incubated at 66 °C [129].

Lin et al. developed a machine learning (ML) model that conducts multi-biomarker analysis utilizing the discriminative feature selection [128]. This approach aims to provide a more accurate, reliable, and minimally invasive method for the detection of lung cancer including mRNA biomarkers [128]. There are four main parts of the detector demonstrated in Figure 4D: control, optic, heating, and moving modules [128]. In Figure 4D, commands are issued by a user, which are further processed by a digital signal processor (DSP) to coordinate the actions of the other three modules [128]. Data processing is accomplished in the control model, that involves regulating the temperature, setting the parameters, and moving the control [128]. There is an incident light path and a fluorescent receiving light path in the optic model [128].

Lithwick-Yanai et al. developed and validated an miRNA-based diagnosis which was performed by qRT-PCR for profiling of miRNAs [104]. As Figure 4E suggests, hsa-miR-375 was used to detect the medullary carcinoma, a form of thyroid cancer in LDA [104]. All fourteen medullary samples, including five indeterminate cases, exhibited high hsa-miR-375 expression in the training set [104]. While in the validation set, all three medullary carcinoma samples were accurately classified as suspicious, although one sample (Bethesda V) did not show hsa-miR-375 overexpression and was not identified as medullary carcinoma [104].

### 2.4. Detection with SERS

In 1974, Fleischmann was the first to observe surface-enhanced Raman scattering (SERS) when they noted a strong Raman signal from pyridine molecules adsorbed on a rough silver electrode [130]. Over the past decade, both Raman and IR spectroscopy have been used in cancer diagnosis by the detection of molecular fingerprints for bladder, breast, lung, and prostate cancer types in blood and serum [131].

SERS is a vibrational spectroscopic method where molecules adsorbed on nanostructured metallic surfaces, including gold and silver nanoparticles, experience enhancement of the Raman signal, relative to the Raman signal of a bulk solid substance, by several orders of magnitude or even more. A significant electromagnetic enhancement from localized surface plasmon resonances and a chemical enhancement from charge-transfer reactions between molecule and metal are two complementary mechanisms, which can lead to average analytical Raman signal enhancements of the order of 10^6^, while maximum enhancement in so called “hot spots” can be as high as 10^14^ when even a single molecule detection/identification is possible. Designed plasmonic nanostructures that strengthen electromagnetic fields to generate “hotspots” significantly increase Raman signals. One of the SERS technique’s main benefits is that it can detect analytes at very low concentrations, sometimes even in complex biological samples, while producing detailed vibrational fingerprints. SERS is a label-free, ultra-sensitive, and non-invasive technique, which operates in ambient conditions and provides immediate molecular and structural data. In addition, the method supports multiplexed identification, which allows the analysis of multiple biomarkers [132].

Some recent applications of SERS substrates have reported a significant improvement in terms of consistency and analytical performance due to a few advances in nanofabrication methods and a more accurate self-assembly processes. Advances in surface-engineering methods, such as the use of self-assembled monolayers and optimized surface chemistries, in many cases have helped to improve assay selectivity and minimize background signals [132,133]. There are still some challenges to a wider application of the SERS technique. For instance, for the clinical application of sandwich SERS immunoassays there is a significant challenge of non-specific binding, including non-specific protein absorption, but these can be decreased by several methods, including application of self-assembled monolayers, better blocking agents, and the application of non-noble metal-made substrates made, for instance, of silicon or aluminum foil, etc., which may also decrease the assay cost and improve selectivity [16,134,135].

In spite of those challenges, SERS is becoming an effective instrument for biomolecular sensing in two modes. The first mode is a direct SERS that operates when an analyte has been directly absorbed on the surface of an SERS substrate or on the surface of SERS active nanoparticles (usually gold or silver NPs) in suspension. The indirect SERS have a sensing molecule like an antibody or aptamer that binds to the analyte molecule and is most commonly represented by sandwich-type immunoassays with SERS detection. In this assay the target biomarker/antigen binds to the capture antibody on a substrate surface first and then a nanotag modified with many (sometimes 1000s) Raman reporter molecules and many capture antibody molecules binds to the same antigen molecule, anchoring the nanotag (usually gold or silver nanoparticles of 40 to 60 nm diameter) to a substrate surface, which provides a highly magnified Raman signal, since 1000s of Raman active reporter molecules are present on the surface of just one nanotag, which may be bound to just one antigen molecule on the surface of the substrate. This method enables the detection of many biomarkers, including cancer biomarkers at sub-picomolar or even femtomolar concentrations. Sandwich SERS immunoassays are therefore among the most sensitive techniques for early cancer diagnosis [136,137].

Publications comprising the clinical performance of the SERS technique in the detection of cancer biomarkers since 2010 are described in Table 4, with most of them reporting sensitivity and specificity values.

The studies revealed the parameters of the average clinical performance by direct and indirect SERS detection, such as sensitivity constituting 90% and 92%, specificity 95% and 90%, and accuracy 95.19% and 98%, respectively, for both methods. Overall performance for both methods showed 91% of sensitivity, 93% of specificity, and 97% of accuracy.

The highest performance for direct SERS was by Hu et al. demonstrating 98.9% of sensitivity and 100% of specificity in the detection of metabolites and exfoliated tumor cells [138]. As for indirect SERS detection, Weng et al. showed comparatively high sensitivity and specificity for the miRNA-155 biomarker in cancer detection [139].

**Table 4:** Clinical detection of cancer biomarkers using SERS.

First Author, Year	Assay/Data Processing Method	Biomarker (Cancer Type)	Cancer Patients/Control Patients	Sensitivity/Specificity/Accuracy
**Direct SERS:**				
Feng, 2017 [140]	Direct SERS, PLS-DA	Urinary modified nucleosides (nasopharyngeal)	62/52	95.2%/97.2%/-
		Urinary modified nucleosides (esophageal)	55/52	90.9%/98.2%/-
Hernandez-Arteaga, 2017 [141]	Direct SERS	Sialic acid in the saliva (breast)	100/106	94%/98%/-
Koo, 2018 [142]	Direct SERS	ERG and PCA3 (prostate)	90/30	87%/90%/-
Paraskevaidi, 2018 [143]	Direct SERS	CA125 (ovarian)	27/28	80%/94%/-
Qian, 2018 [144]	Direct SERS	Proteins and nucleic acids (lung)	61/66	95.1%/100%/-
Hernadez-Arteaga, 2019 [145]	Direct SERS	Sialic acid (breast)	35/129	80%/93%/-
Lin, 2019 [146]	Direct SERS	Modified nucleoside (gastric)	50/48	84%/95.8%/-
		Modified nucleoside (breast)	43/48	87.5%/87.5%/-
Moisoiu, 2019 [147]	Direct SERS	Anionic purine metabolites: uric acid, xanthine, and hypoxanthine (breast)	53/22	81%/95%/-
Perumal, 2019 [148]	Direct SERS	Hp (epithelial ovarian cancer)	54/57	94%/91%/-
Lin, 2020 [149]	Direct SERS	Tumor markers such as DNA, RNA, proteins, etc. (gastric)	50/48	90%/93.8%/-
		Tumor markers such as DNA, RNA, proteins, etc. (breast)	43/48	96%/93.8%/-
Lin, 2020 [150]	Direct SERS	DNA, RNA, proteins (colorectal)	63/53	95.8%/94.3%/-
Ma, 2021 [151]	Direct SERS	Urinary metabolite (prostate)	12/63	86%/87.1%/-
Hu, 2021 [138]	Direct SERS	Metabolites, exfoliated tumor cells (bladder)	161/87	100%/98.9%/-
Nargis, 2021 [152]	Direct SERS	SERS features related to DNA, proteins, and lipids (breast)	17/12	90%/98.4%/94%
Xiong, 2023 [153]	Direct SERS	L-tyrosine; acetoacetate, riboflavin; phospholipids, amide-I, alpha-helix (bladder; adrenal; acute myeloid leukemia)	80/30	-/-/98.27%
Arithmetic mean				90%/95%/96%
Median				90%/95%/96
**Indirect SERS:**				
Banaei, 2021 [154]	SERS immunoassay	EVs (pancreatic)	5/5	95%/96%/-
Han, 2022 [155]	Microfluidic SERS	CD63, VIM, EpCAM (osteosarcoma)	20/20	100%/90%/95%
Weng, 2022 [139]	SERS with CHA amplification	miRNA-21 (breast)	30/30	93.3%/100%/100%
		miRNA-155 (breast)		100%/100%/100%
Murali, 2023 [156]	Indirect SERS (using nanotags)	ER, PR, HER2 (breast, singleplex)	N/A	95%/92%/-
		(duplex)		88%/85%/-
		(triplex)		75%/67%/-
Arithmetic mean				92%/90%/98%
Median				95%/92%/100%
**Direct and Indirect SERS Combined:**				
Arithmetic mean (overall):				91%/93%/97%
Median (overall):				92%/94%/98%

Unfortunately, most of these studies did not include calculations of the accuracy value, one of the most important clinical parameters, making the selection for this value a bit scarce. Nevertheless, this is enough to make relevant conclusions about the development of the SERS method’s performance in clinical detection of various cancer biomarkers. Some recent multi-modal approaches still report accuracy values and demonstrate how combining detection methods can enhance analytical performance. For example, a study using hexoctahedral Au nanoparticles shows how combining SERS and fluorescence can greatly improve cancer detection [157]. The dual-modal bioprobes provided highly sensitive SERS (10^−12^) and clear fluorescence imaging of breast cancer cells [157]. The method showed 94% accuracy in identifying breast cancer subtypes, indicating that multiplexing both techniques can be beneficial for selectivity and sensitivity [157]. Similarly, another study created a multi-modal technique combining ATR-FTIR and SERS to analyze blood-based RNA biomarkers from breast cancer patients [158]. Zhang et al. (2024) determined the main molecular fingerprints and applied several ML algorithms, achieving a blind test accuracy of 92%, specificity of 95%, sensitivity of 96%, and an F-score of 95% [158].

**Figure 5:**
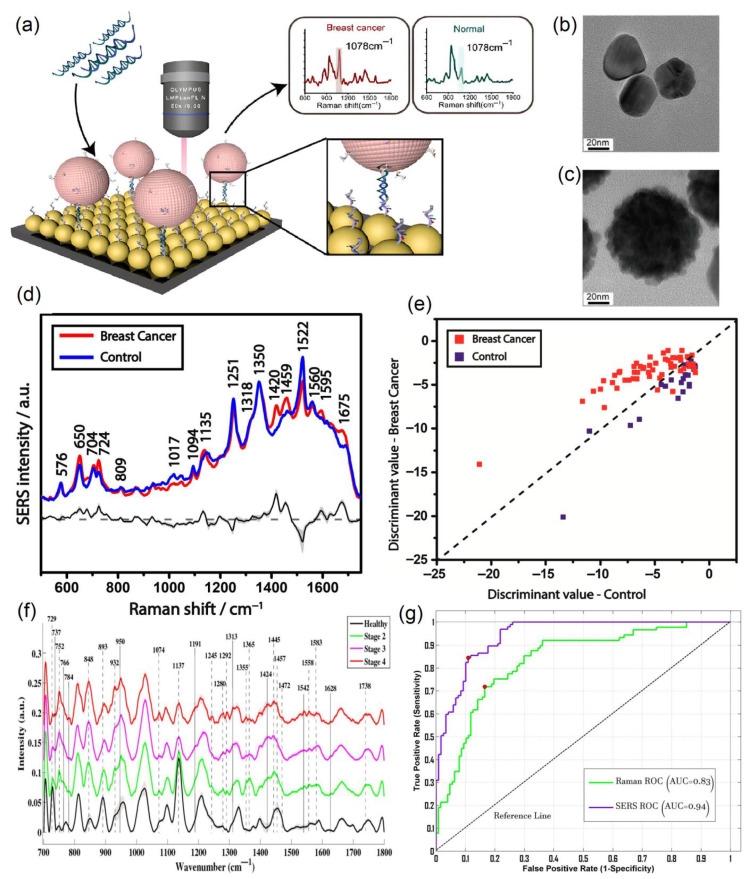
Clinical performance of the direct SERS technique using various data analysis methods for breast cancer. (**a**) Schematic illustration of the SERS biosensor for the detection of miRNA with Raman spectra corresponding to breast cancer patients and healthy controls. TEM images of (**b**) Ag NPs and (**c**) Ag@4-MBA@Au NPs, reproduced with permission from Weng et al. [139] © 2022 Elsevier B.V. (**d**) Average SERS spectra for breast cancer patients (red) and control samples (blue), as well as their difference spectrum (black) and standard deviation (gray shaded area). (**e**) PCA-LDA model for breast cancer and control samples, reproduced with permission from Moisoiu et al., Applied Sciences, published by MDPI, 2019 [147]. (**f**) Average SERS spectra for control samples and different stages of breast cancer. (**g**) Comparison of ROC curves for regular Raman spectroscopy (green) and SERS (purple) techniques for breast cancer, reproduced with permission from Nargis et al. [152] © 2021 Elsevier B.V.

Figure 5 contains the representations from articles in which the clinical performance of SERS was presented. In Figure 5a the SERS biosensor was prepared using a two-dimensional Au@DNA-Si chip substrate and Au@4-MBA@Ag@DNA NPs by additionally coupling to the catalytic hairpin assembly (CHA).

Weng et al. obtained good clinical parameters in the detection of miRNA-21 and miRNA-155, with 93% sensitivity, 100% specificity, and 100% accuracy for the former, and 100% sensitivity and the same specificity and accuracy for the latter [139].

In order to picture an idea of the morphology of silver nanoparticles (Ag NPs) and core–shell NPs (Ag NPs combined with 4-MBA with an outer Au layer (Ag@4-MBA@Au)), Transmission Electron Microscopy (TEM) was performed, the results of which are presented in Figure 5b,c. The surface of the core–shell NPs differs from the relatively smooth surface of the Ag NPs due to the fact that the Au shell was deposited on the surface of the Ag@4-MBA@Au by applying lots of gold nanoparticles, increasing its roughness to enhance the SERS signal.

Clinical detection using the SERS technique was mostly performed by directly applying this method, i.e., without any tags or labels [147]. Research made by Moisoiu et al. in 2019 [147] utilizes direct SERS clinical detection for breast cancer. For their study, Moisoiu et al. collected a total of 75 urine samples, 53 of which corresponded to breast cancer patients (stage I–3, stage II–21, stage III–29) and the remaining 23 were control samples. The average SERS spectra for both breast cancer and control samples are illustrated in Figure 5d, along with the difference in spectra and corresponding standard deviation. The authors concluded that adding Ca^2+^ or Mg^2+^ cations to AgNPs enhances the chemisorption of anionic species, namely uric acid, xanthine, and hypoxanthine, which increases their Raman intensities, while not causing nanoparticle aggregation. Moisoiu et al. used a 532 nm excitation laser since it is relatively close to the UV–Vis absorption maximum of the silver nanoparticles. They additionally performed UV–Vis measurements of the hya-AgNPs with and without Ca^2+^ ions and confirmed that there were negligible alterations between two spectra. Moisoiu et al. performed the principal component analysis–linear discriminant analysis (Figure 5e) and used it as the statistical model to analyze their SERS spectral data. Moisoiu et al. achieved a sensitivity of 81%, specificity of 95%, and accuracy of 88% as a result of their research [147].

Another clinical application of direct SERS analysis for breast cancer was accomplished by Nargis et al. in 2021 [152]. In their research, seventeen breast cancer serum samples (seven, five, and five samples for Stage 2, Stage 3, and Stage 4, respectively) and twelve control serum samples were used to obtain SERS spectral data for further analysis. Figure 5f demonstrates the average spectral data for the SERS measurements of this study, in which the healthy group is compared to Stage 2, 3, and 4 breast cancer samples. They made an observation that, generally, serum samples of breast cancer patients contain higher concentrations of DNA bases, proteins, lipids, etc., in comparison with control samples. Along with the SERS measurements, Nargis et al. made regular Raman measurements and compared these two methods in terms of their performance. It can be seen from Figure 5g that the area under the receiving operating characteristic (ROC) curve is noticeably better for the SERS method (0.94) than for Raman spectroscopy (0.83). Finally, Nargis et al. obtained good clinical parameters for their study—90% sensitivity, 98.4% specificity, and 94% accuracy—which correspond to the SERS technique, while the same parameters for regular Raman spectroscopy were worse (88.2%, 97.7%, and 83.4%, respectively). In other words, SERS is a much more suitable technique for breast cancer diagnosis than regular Raman spectroscopy [152].

Referring again to Table 4, there is a significantly larger number of clinical studies made using direct SERS, rather than SERS using different kinds of nanotags. For papers applying the SERS technique directly, arithmetic mean and median values do not differ much from each other. For the clinical research articles using indirect applications of SERS, however, the difference between arithmetic mean and median numbers is more noticeable. In addition to that, since the size of the selection is rather small (four research papers) and the range for some clinical parameters is large (e.g., 33% for specificity), it would be more appropriate to take into consideration the median values rather than arithmetic mean values in this case. Thus, the median sensitivity for studies using the direct SERS technique is noticeably smaller in comparison with indirect SERS applications (90% and 95%, respectively). For median specificity the trend is opposite—clinical papers utilizing the SERS method directly show better values for this parameter than articles based on clinical SERS detection with nanotags (95% against 92%). Use of various nanotags in SERS detection increases the sensitivity of the method due to a large increase in the intensity of the Raman signal; however, it sacrifices its specificity instead, which can be explained by the non-specific binding [135].

### 2.5. Detection with FTIR

FTIR represents a vibrational spectroscopy technique used to identify and quantify molecules through the absorption of infrared light by chemical bonds. IR radiation excites specific functional groups at characteristic frequencies that correspond to vibrational transitions, producing a molecular “fingerprint” [159].

Since FTIR spectra have relatively narrow absorption peaks with a typical width of 10 to 20 cm^−1^, tens of IR peaks can be resolved without a high chance of peak overlap in the IR spectra, which are typically in about the 600 to 4000 cm^−1^ range. That gives FTIR and Raman spectroscopy, which also have relatively narrow peaks of about the same width, a significant multiplexing advantage over fluorescence and UV–vis spectrophotometry, where peak widths are typically in the range of 50–150 nm. However, the whole visible range spans only from 400 to 780 nm, therefore absorption/florescence peaks from several/multiple analytes would have a high chance of overlapping, making multiplexing in UV–vis range spectroscopy difficult and seldom possible beyond duplexing or triplexing. On the contrary, dozens of functional groups in a multitude of organic compounds can be resolved in FTIR spectra, making reliable identification and often quantification of those compounds possible at moderate concentration (about ppm or microgram level and above) but not at a very low concentration, such as ppb level, where the analyte is frequently detected by MS, fluorescence, SERS, etc. FTIR is better than traditional, dispersive IR spectroscopy primarily because of the fast time of spectra acquisition (seconds or less vs. 5–15 min), which often enables FTIR to take many (100+) spectra and average them with a large improvement in signal-to-noise ratio in significantly less time than that required for an acquisition of a single dispersive IR spectrum. Therefore, by far, most of the IR spectrometers produced nowadays are FTIR spectrometers. FTIR spectrometers are relatively compact benchtops, similar to the size of a microwave oven or less, that have a moderate price of about 10–15 K USD by order of magnitude, which is about one order of magnitude less than the minimum price of mass spectrometers.

FTIR can be operated in several measurement configurations, including transmission, attenuated total reflectance (ATR), and diffuse reflectance modes [160]. These modalities enable the analysis of liquids, solids, powders, thin films, and a wide range of biological specimens with minimal or no sample preparation. Among them, ATR-FTIR has gained particular significance in biomedical and clinical applications as it permits the direct measurement of biological fluids and complex matrices by pressing the sample against a high-refractive index crystal [161]. This approach minimizes sample handling, reduces optical path length variability, and improves reproducibility, making ATR the preferred technique for diagnostic spectroscopy. In FTIR instruments, the Michelson interferometer is typically used to collect the interferogram [162] and the beam splitter divides the incoming IR beam into two paths directed toward a fixed and a movable mirror. After reflection, the beams recombine at the beam splitter, generating an interference pattern that is detected by a photodetector. A Fourier transform is then applied to convert the interferogram into an infrared spectrum [163]. Historically, the development of Fourier-transform spectroscopy was advanced in the early 1950s by John Strong and his team at Johns Hopkins University. They constructed and utilized far-infrared spectrometers [164]. Over the following decades, the potential of FTIR for analyzing biological samples, including cancer detection, has become widely recognized [165].

A number of studies have investigated the role of spectroscopy for the rapid diagnosis of brain cancer present in differing disease states. Values of sensitivity (93.2%) and specificity (92.8%) were higher for Si IREs than the values from the 433 retrospective patient cohort by Smith et al. [166]. The obtained results illustrate a step in the translation of ATR-FTIR into a clinical application. More cancer-related clinical FTIR studies are summarized in Table 5.

It is known that measures like sensitivity, specificity, and accuracy are significant parameters in assessing the evidence of screening tests [181].

In terms of sensitivity/specificity/accuracy, FTIR demonstrated promising results. For example, Paraskevaidi et al. used attenuated total reflection Fourier-transform infrared (ATR-FTIR) spectroscopy to analyze urine samples from women with endometrial (*n* = 10) ovarian cancer (*n* = 10), as well as from healthy individuals (*n* = 10). By combining ATR-FTIR with different multivariate and statistical models, they achieved good accuracy for both endometrial (sensitivity: 95%, specificity: 100%, accuracy: 95%) and ovarian cancer (sensitivity: 100%, specificity: 96.3%, accuracy: 100%) [169]. The enhancement of these values can lead to improvements of the outcome in the detection of various cancer types.

SERS and FTIR are both vibrational spectroscopy techniques, which have their own advantages and disadvantages, hence it would be appropriate to compare these two methods in terms of their clinical performance. If we compare these average calculated parameters separately, the difference between FTIR and SERS is somewhat noticeable; however, overall, there is just a slight advantage of SERS over FTIR and, in general, their clinical performance is rather comparable.

To the best of our knowledge, there are just several reported applications of SEF for the detection of cancer biomarkers with a relatively broad range of reported sensitivities/LODs, and we could not find any clinical reports of SEF for cancer diagnostics.

**Figure 6:**
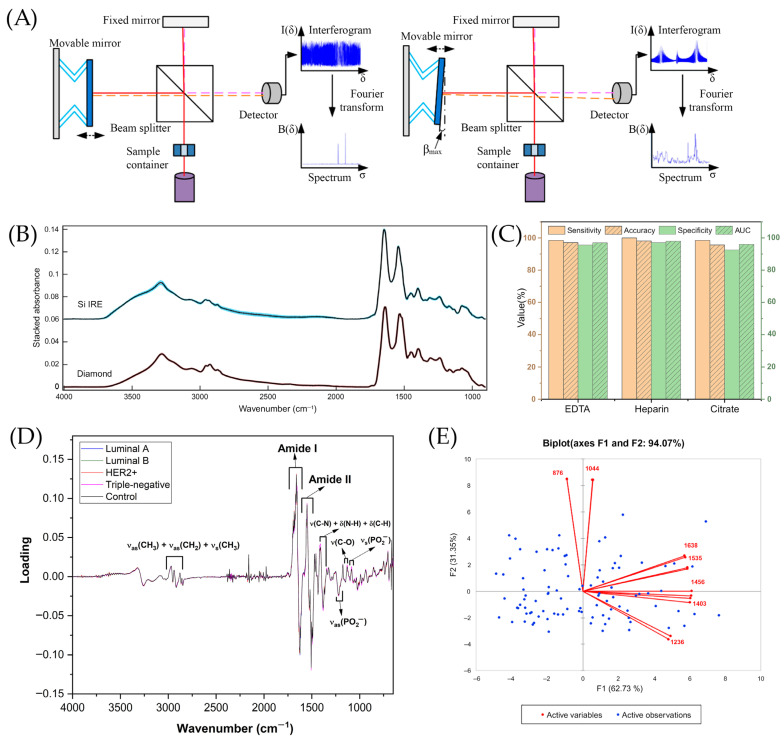
The performance of FTIR in the detection of cancer diseases. (**A**) Schematic illustration for a Michelson interferometer, adopted with permission from Chai et al. [163] under the Creative Commons Attribution 4.0 International License. (**B**) Silicon (Si) internal reflection element (IRE) ATR-FTIR in human serum with Si shown in blue and diamond shown in red [171], adopted with permission from Butler et al. [171] under the Creative Commons Attribution 4.0 International License. (**C**) The performance of principal component linear discriminant analysis (PC-LDA) for late cancer patients and healthy controls (HC) with EDTA, heparin, and citrate [177], adopted with permission from Chen et al. [177] Copyright © 2022 Elsevier V.B. (**D**) The representation of five models’ loadings for orthogonal partial least squares discriminant analysis (OPLS-DA) [179], adopted with permission from NMP de Souza et al. [179] Copyright © 2023 Elsevier V.B. (**E**) Principal component analysis (PCA) plots for malignant and benign samples [172], adopted with permission from Bangaoil et al. [172] under the terms of the Creative Commons Attribution License.

Butler et al. designed disposable silicon (Si) internal reflection element (IRE) slides that provide rapid access to the preparation and analysis of several samples, making ATR-FTIR more suitable for clinical diagnosis in blood serum [171]. The spectra of Si IRE was compared to the conventional method with diamond IRE to analyze the human blood serum by removing the biological variation [171]. There is a large standard deviation between 2700 and 2000 cm^−1^ wavenumbers and there are only a few differences in bands such as the N-H protein band that is between 3500 and 3300 cm^−1^ and between peaks of amide I and amide II as shown in Figure 6B [171].

Chen et al. performed transmission FTIR to detect esophageal squamous cell carcinoma (ESCC) in plasma and serum using barium fluoride (BaF_2_) [177]. As shown in Figure 6C, on each anticoagulant, every model reached more than 95% for sensitivity, specificity, and accuracy parameters using PC-LDA, indicating the possibility to distinguish late cancer patients from healthy controls using plasma or serum samples [177].

As for Figure 6D, the study designs a method for ATR-FTIR, coupling it with chemometric methods to distinguish the subtypes of BC at the molecular level in several blood plasma samples [179]. The loadings of one latent variable assisted in the differentiation of different molecular subtypes and cancer stages, since each molecular subtype exhibits distinct metabolomic profiles and cancer stages that are more associated with variations in the concentration of specific analytes [179].

Bangaoil et al. assessed the potential of ATR-FTIR as a supplementary technique for the examination of H&E-stained tissues for diagnosing lung cancer [172]. In Figure 6E, F1, one of the principal components, accounted for 62.73% of the variance due to amides, lipids, and nucleic acids with positive loadings at 1638 cm^−1^ and 1535 cm^−1^, likely to elevate the levels of β-sheet protein structures [172]. F2, constituting 31.35% of the variance, was mostly associated with carbohydrates at 1044 cm^−1^ and phosphorylated proteins at 876 cm^−1^, according to Figure 6E [172].

## 3. Clinical Applications for Cancer Biomarker Detection

In total, in the clinical section, we analyzed 126 clinical articles with reported sensitivity, specificity, and other figures of merit, including the area under the receiver operating curve (AUC) values. For each technique, we calculated the average and median sensitivity, specificity, and AUC values and compared them across different techniques (e.g., mass spectroscopy, ELISA, SERS, etc.) The results are summarized in Table 6. From Table 6, sensitivity (%) is an ability of the method to detect subjects with a disease (e.g., specific type of cancer). The specificity (%) is an ability of the method to obtain negative results for a subject who does not have a disease [182]. The area under the receiver operating curve (AUC) defines the overall accuracy of the test in classifying individuals with and without a disease or condition. The greater the AUC, the better the discriminatory power is. An AUC of 0.5 means no discrimination, 0.7–0.8 is considered acceptable, and >0.9 is outstanding [183]. The comparison of all three parameters together gives a broad picture of the clinical performance of various methods. Across all clinical samples, the average number of patients ranged from 95, the lowest, in MS up to 296, the largest, in ELISA and IA methods, which are calculated for comparison between techniques in Table 1, Table 2, Table 3, Table 4 and Table 5. Overall, for this review about clinical detection of cancer biomarkers among the 121 studies represented in these tables, where number of patients were clearly indicated, the average number of patients is 163, while the median is significantly lower at 106 patients.

Based on average sensitivity and specificity, among reported clinical diagnostics studies, SERS and FTIR have better results (91.5%) than mass spectroscopy (87%), PCR (83.5%), ELISA (80.5%), and immunoassays (79%). Considering each of the two parameters separately, we observed a similar trend. Based on sensitivity alone, FTIR has a better performance (94%) than SERS (88%), mass spectroscopy (88%), immunoassays (84%), PCR (83%), and ELISA (77%). In terms of specificity, we observed slightly different trends, SERS being slightly better (93%) than FTIR (89%) and mass spectroscopy (86%). PCR and ELISA demonstrated comparatively similar specificity (84%) with a median specificity value of ELISA (88.4%) being slightly better than that of PCR (83.8%). From average accuracy, ELISA and SERS (97% and 95%, respectively) demonstrated slightly better results than FTIR (91%), and even better results than mass spectroscopy (89%), PCR (84%), and immunoassays (77%). Based on the average AUC, SERS demonstrated better results than FTIR (AUC 0.97 and 0.95, respectively), and even better results than mass spectroscopy and PCR (0.89), ELISA (0.83), and immunoassays (0.76).

Overall, considering all the parameters, the order, in terms of the clinical performance of the six methods discussed in this section and based on the literature tabulated in the tables above, is the following: (1) SERS, (2) FTIR, (3) mass spectroscopy, (4) PCR, (5) ELISA, and (6) immunoassays.

## 4. Challenges and Perspectives

There are several challenges that limit the applications of both direct and indirect detection techniques in clinical diagnostics. The non-specific binding is a common issue for most immunoassays, as can be seen from the comparatively low average specificity from immunoassay-based studies, including chemiluminescent, 3D immunoassays, and electro-chemiluminescent and fluorescent immunoassays [16,184]. Protein non-specific binding is formed on the gold surface, for instance, due to the formation of a sulfur–gold dative bond. Modifications of gold substrates could be the solution to the problem of non-specific binding, for example, using blocking agents or protective monolayers that prevent the formation of such a limitation [136].

In addition, there are some perishable products used in indirect methods of detection that require special conditions for shipment and storage. For example, they need to be stored at low temperatures which vary from −20 to −40 C. Methods like PCR and ELISA may result in financial losses due to the nature of perishable products [185]. There is significant variability in the binding efficiency of antibodies associated with their pharmacokinetics (PK). Such properties include different binding affinity, binding specificity, cytotoxicity, stability, and others, and they significantly affect the successful applications of antibodies in research and result in poor PK [186]. It was observed by Luo et al. that 13% impurity significantly decreases the sensitivity and binding efficiency of recombinant antibodies, despite the fact that the general purity of these antibodies was expected to be equal to 98.7% [187]. Lot-to-lot or batch-to-batch variances affect the results, decreasing the accuracy of fabricated methods, which increases the uncertainty of the experiments [187].

Despite these limitations, direct methods such as SERS and FTIR demonstrated comparatively high accuracy, sensitivity, and specificity parameters, since these methods do not depend on the sensing molecules (antibodies and aptamers). There are advances in machine learning (ML) for clinical diagnosis of cancer biomarkers that may accelerate the commercialization.

Artificial intelligence (AI) enables devices to mimic human behavior and efficiently process datasets; machine learning is an example of how AI is applied, enabling computers to learn without requiring explicit programming [188]. It has also been applied in cancer diagnostic research for pancreatic, bladder, nasopharyngeal carcinoma, and breast cancer [188]. In certain cases, it achieved a performance comparable to human expertise [188]. Along with the integration of histopathology workflows and advances in machine learning, algorithms that predict biomarker status from hematoxylin and eosin (H&E)-stained tissue, along with other cancer types, have started gaining attention in this research area [189]. This approach offers a potentially more efficient and available alternative to immunohistochemistry, as well as opening up the opportunity to identify tissue morphology that correlates with biomarker status [189].

**Figure 7:**
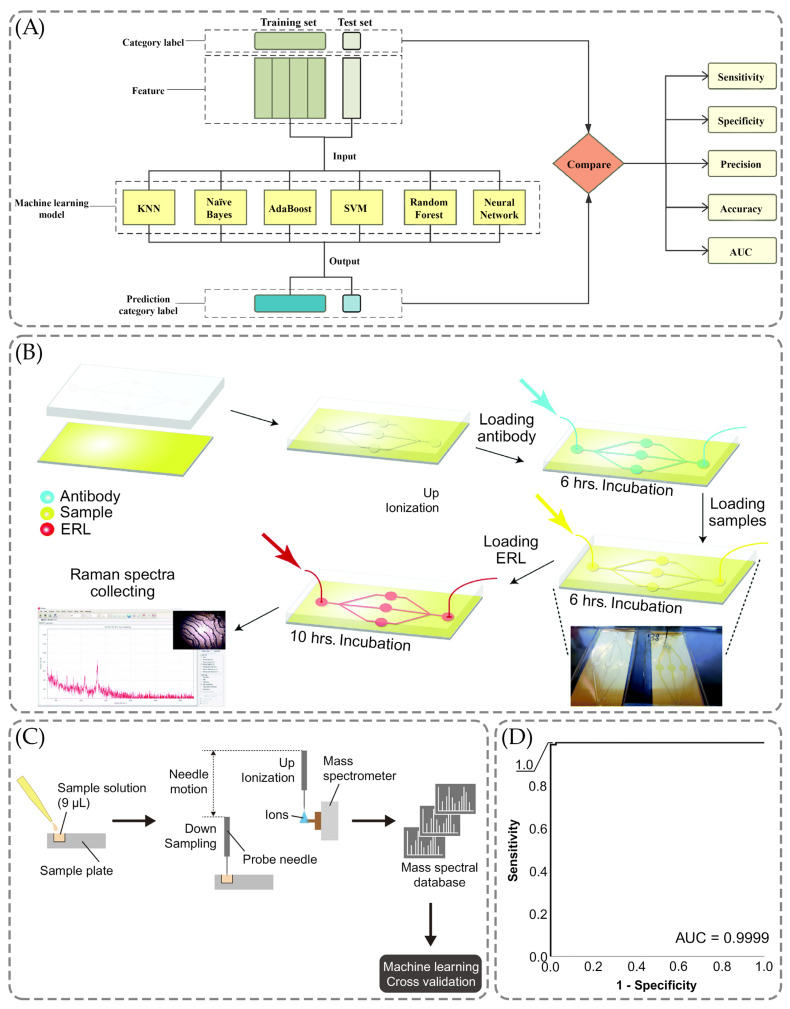
The application of the ML methods for the cancer biomarkers detection. (**A**) The scheme of the use of ML for the early detection models of the lung tumor, adopted with permission from Xie et al. [188] under the Creative Commons Attribution 4.0 International License. (**B**) The schematic illustration of a microfluidic immunoassay using SERS for the multiplexed detection of human epididymis protein 4 (HE4), carbohydrate antigen (CA19-9), mesothelin, matrix metalloproteinase-7 (MMP7), and mucin-4 (MUC4) in serum fluid, reused with permission from Banaei et al. [190] under the Creative Commons Attribution 4.0 International License. (**C**) Probe electrospray ionization-mass spectrometry (PESI-MS) in the application with ML, adopted with permission from Kiritani et al. [193] under the Creative Commons Attribution 4.0 International License. (**D**) The graph obtained by discriminant analysis for receiver operating characteristic curve, adopted with permission from Kiritani et al. [193] under the Creative Commons Attribution 4.0 International License.

Table 7 presents the average clinical performance of the biosensors working principle, which includes different methods related to machine learning (ML). Average sensitivity was equal to 93%, specificity was equal to 96.7%, accuracy was equal to 93.2%. The AUC was equal to 0.945. Overall, the average calculated AUC in ML-based cancer biomarker detection assays is above average the AUC calculated in Table 6 for MS-, PCR-, ELISA-, and IAs-based cancer biomarker detection methods. The examples of successful application of ML methods in cancer biomarker detection are shown in Figure 7 below.

However, it is about the same as the AUC of FTIR (0.95) and a bit lower than the AUC of reported SERS (0.97) assays for clinical cancer detection. Aitekenov et al. showed the same trend in terms of which SERS performance proved to be better in terms of sensitivity and other analytical parameters in comparison with FTIR [196]. 

Xie et al. aimed to investigate the metabolomics of lung cancer and its biomarkers, differentiating histology and the stages of the illness and offering a novel approach to using machine learning to improve the diagnostic approach of the detection of the early stage of this cancer [188]. As illustrated in Figure 7A, there were six ML models: Naïve Bayes, AdaBoost, K-nearest neighbor (KNN), support vector machine (SVM), Random Forest, and Neutral Network [188]. The prediction model was also constructed using the training set with stage I patients and healthy individuals, respectively, 43 and 35 [188]. The results have indicated the better performance for Neutral Network and Naïve Bayes compared to the other four methods, with specificity, sensitivity, accuracy, and AUC parameters reaching 100% [188].

Similarly, the multiplex detection using SERS was also performed by Banaei et al. using the microfluidic chip and targeting the detection of human epididymis protein 4 (HE4), carbohydrate antigen (CA19-9), mesothelin, matrix metalloproteinase-7 (MMP7), and mucin-4 (MUC4) [190]. They proposed that the multiplex detection allows one to improve the specificity of the detection of cancer biomarkers and the microfluidic chip allows one to enhance the immunoassay reproducibility [190]. Figure 7B shows the use of the chip to ensure the flow on-chip was followed by injections [190]. The substrate was coated with Dithiobis-succinimidyl propionate (DSP) and left for reaction with the antibody throughout six hours, forming the layer of capture antibodies [190].

Figure 7C demonstrates the workflow of the probe electrospray ionization-mass spectrometry with ML methods to obtain mass spectra and further analysis of them using logistic regression [193]. As shown in Figure 7C, the solutions were constituted of nine microliters of the sample to conduct PESI-MS, combining the ambient ionization unit and triple quadrupole MS [193]. The results were 99, 100, and 99% for sensitivity, specificity, and accuracy parameters along with the area under the curve achieving the 0.9999 value in Figure 7D [193].

## 5. Conclusions

There is a variety of sensing techniques that are applicable and successful in the detection of cancer biomarkers. A comparative quantitative analysis of the major performance parameters reported in 116 publications demonstrated that direct spectroscopic sensing methods (both SERS and FTIR) demonstrated slightly better or comparable order of clinical performance parameters in comparison with other techniques (MS, PCR, IAs, ELISA detecting methods). However, based on the limited number of available publications, we observed some edge in the reported area under the curve (AUC) for the direct spectroscopic methods such as SERS (AUC 0.97) and FTIR (AUC 0.95) in comparison with immunoassays, ELISA, MS, and PCR (AUCs in 0.76 to 0.89 range).

Despite multiple reports about the validation of both techniques in large patient groups, challenges in the standardization of sample collection, storage and preparation procedure, and standardization of data acquisition and processing of spectral data need to be addressed for successful implementation of them in clinical practice [165]. There is an increasing number of studies about applications of machine learning for cancer diagnostics and this approach to data analysis may render substantial improvement in sensitivity and specificity of clinical cancer diagnostics as well as potentially decrease the time and even cost of the clinical analysis for cancer markers, making cancer more detectable at early stages of the disease and therefore making it more curable. The key challenge in early cancer diagnostics lies in translating ultra-sensitive in vitro laboratory technologies into practical tools that can operate reliably in clinical settings when faced with real, complex biological samples. Integrating machine learning and AI will further enhance signal interpretation, enabling more accurate detection in early-stage patients, while the development of non-invasive, low-cost diagnostic platforms will expand monitoring capacity and make early detection accessible to a broader population.

## Figures and Tables

**Table 1:** Clinical studies on human body fluids for cancer biomarkers detection using tandem mass spectrometry methods.

Author, Year	Technique	Analyte	Detection	Sensitivity	Specificity	Number of Samples	Other FOM
Takayama, 2016 [32]	UPLC-MS/MS	Polyamines	Breast cancer	80%	80%	172 (111+, 61−)	
Mu, 2016 [33]	SELDI-TOF-MS	Urinary glycopeptides	Endometrial, ovarian, and cervical cancer	100%	91.70%	12 (4 endometrial cancer, 4 ovarian cancer, 4 cervical cancer)	
Xiao, 2016 [34]	TMT, LC-MS/MS, ELISA	Proteins (cystatin B (CSTB), triosephosphate isomerase (TPI1), and deleted in malignant brain tumors 1 protein (DMBT1).	Gastric cancer	85%	80%	80 (40−, 40+)	Accuracy: 0.93; AUC: 0.93; ROC: 0.81–0.92, *p* < 0.05
Zhong, 2016 [35]	UPLC-MS	Metabolome (LysoPC 18:1)	Breast cancer	77.80%	100%	55 (30+, 25−)	AUC: 0.93; 95% CI: 0.836–1.000
Chen, 2017 [36]	LC-MRM/MS	56 salivary proteins	Oral cancer	54.1–86.9%	74.1–91.4%	119 (58−, 61+)	AUC: 0.59–0.91; Cut-off value: 2.21–6798.48 ng/mL
Fujita, 2017 [37]	LC-MS/MS, Western blot	FABP5	Prostate	60%	100%	18 (6 negative biopsy, 6 Gleason score 6 prostate cancer, 6 Gleason score 8–9 prostate cancer)	AUC: 0.856
Hirata, 2017 [38]	GC-MS/MS	16 targeted metabolites	Pancreatic cancer	90.70%	89.50%	1st set: 113 (58+, 55−), 2nd set: 32 (16+, 16−)	AUC: 0.931
Chen, 2019 [9]	UPLC-QTOF/MS	6 metabolites	Laryngeal cancer	95%	97%	66 (29−, 37+)	AUC: 0.97
Zhou, 2020 [39]	LC-MS/MS	11 proteins	Early gastric cancer	66.70%	86.70%	30 (15−, 15+)	AUC: 0.796
Arendowski, 2020 [40]	LDI-MS	Compounds, lipids	Kidney cancer	74%	78%	100 (50−, 50+) but sensitivity 37 sample, specificity 39 sample, accuracy 32 sample	Accuracy: 76%; AUC: 0.669
Assad, 2020 [41]	LC-QTOF MS	Metabolome (glycerophospholipids and oligopeptides)	Breast cancer	65.20%	77.10%	58 (23+, 35−)	AUC: 0.732
Bian, 2020 [30]	DIAAA derivatization-UHPLC-QTOF/MS	605 carboxylic acids	Colorectal cancer	96.90%	94.40%	Training cohort (43 CRC and 32 healthy human serum), and validation cohort (15 CRC and 15 healthy human serum)	AUC: 0.85; Cut-off 0.71
Cheng, 2020 [42]	Q-TOF-MS	Phosphatidylcholine and phosphatidylethanolamine	Cervical cancer	95.20%	86%	71 CC patients (*n* = 21), squamous intraepithelial lesions (SIL) (*n* = 30), and HC, *n* = 20, 72 = 25 CC patients, 27 SIL patients, 20 HC	AUC: 0.966
Deutsch, 2020 [43]	qMS	Proteome	Pancreatic cancer	90%	90%	31 (16−, 15+)	AUC: 0.91
Xiong, 2020 [44]	LC-MS/MS and SWATH	Bile acids (8 metabolites)	Pancreatic cancer	100%	100%	34	AUC: 1; 95% CI: 1.0
Yang, 2020 [45]	LC–MS/MS	18 proteins	Breast cancer	83.90%	89.70%	114 (51−, 63−)	AUC: 0.901; 95% CI: 0.840–0.962; cut off value 0.736.
Markin, 2020 [31]	GC-MS	Prostate-specific antigen, sarcosine	Prostate cancer	92.60%	91.70%	79 (36, 16, 27)	Accuracy: 87.3%
McKitterick, 2021 [46]	LC-MS/MS	NLLGLIEAK and ELPLYR peptide	Lung cancer				Accuracy: 87.3 ± 41.4%
Chen, 2021 [47]	LC-MS	fecal metagenome and serum metabolome	Colorectal cancer	83.50%	84.90%	44 (11−, 33+)	AUC: 0.92
Gong, 2021 [48]	LC-MS/MS	ICAM1, APMAP	Colorectal cancer	84%	76%	40 (20−, 20+)	AUC: 0.896, 0.854
Jain, 2021 [49]	LC-MS	Proteomics	OSCC	100%	77.60%	99 (49−, 50+)	AUC: 0.94
Kozar, 2021 [50]	HPLC-TQ/MS	4 endogenous metabolites	Endometrial cancer	94%	75%	36 (21−, 15+)	AUC: 92.5%; CI: 90.5–94.5%
Wang, 2021 [51]	GC-MS	L-serine, myo-inositol, and decanoic acid + tPSA	Prostate cancer	82.90%	72.70%	79 (38−,41+)	AUC: 0.781 (*p* < 0.05)
Zhang, 2021 [52]	HILIC-UPLC-HRMS	10 amino acids	Thyroid cancer	91.20%	85.20%	122 (61−, 61+)	AUC: 0.936; R^2^ > 0.99; recover: 92.2–110.3%
Frantzi, 2022 [53]	CE-MS	19-biomarker model	Prostate cancer	87%	65%	147	AUC: 0.81
Lin, 2022 [54]	UHPLC-MS/MS	8-oxodG and 8-oxoG	cervical carcinoma	77.10%	90%	194 (100−, 70+, 24 one-year follow-up)	AUC: 0.871; cut-off value 1.239
Zou, 2022 [55]	LE-ESSI FTICR-MSI	Phosphatidylcholine (32:0) and phosphatidylcholine (34:3)	Gastric cancer	100%	100%	60 (30−, 30+)	AUC: 1.00
Alustiza, 2023 [56]	Mag-HSAE-TD-GC–MS	p-Cresol, 3(4H)-dibenzofuranone,4a,9b-dihydro-8,9b-dimethyl- (3(4H)-DBZ)	Colorectal cancer	87%	79%	80 (32−, 24+, 24 polyps)	AUC: 0.86; 95% CI: 0.75–0.96
Chang, 2023 [57]	UHPLC-MS/MS	17 nucleosides and CRE	Bladder cancer			219	Accuracy: 91–114%; AUC: 0.819: RSDs < 10%
Han, 2023 [58]	UHPLC-MS/MS	19 amino acids and 7 amino-containing neurotransmitters	Gastric cancer	91.40%	87.50%	25 (15+, 10−)	Accuracy: 95%
Li, 2023 [59]	GC-HRMS	8 volatile organic compounds (VOCs)	Breast cancer	76%	92.30%	168 (88−, 80+)	AUC: 0.91; Accuracy: 84.3%
Ossolinski, 2023 [60]	LDI-MSI/MS	10 metabolites	Bladder cancer	83%	100%	6	AUC: 0.944
Sani, 2023 [61]	LC-MS/MS	21 VOCs	Lung cancer	96%	86%	386 (95−, 291+)	AUC TG-7, TG-21: 0.611
			Average/Median Total:33 references	86.23% 87.00%	86.92% 87.50%		

Abbreviations: QTOF—quadrupole time-of-flight, Mag-HSAE—magnetic headspace adsorptive extraction, TD—thermal desorption, SWATH—sequential windowed acquisition of all theoretical fragments, CE—capillary electrophoresis, LE-ESSI—liquid extraction–electrosonic spray ionization, FTICR—Fourier-transform ion cyclotron resonance, HILIC—hydrophilic interaction liquid chromatography, CI—confidence interval, LDI—laser desorption/ionization, SELDI—surface-enhanced laser desorption/ionization.

**Table 2:** ELISA and immunoassay-based detection for the cancer diagnosis.

Author, Year	Analytical Technique	Analyte	Cancer Type	# Sample	Sensitivity, %	Specificity, %	Other FoM (e.g., AUC, Cut-Off Value, etc.)
**ELISA**							
Xiao, 2016 [34]	ELISA	Proteins (cystatin B(CSTB), triosephosphate isomerase (TPI1)	Gastric cancer	40 (20+, 20−)	85	80	ROC: 0.81–0.92; *p* < 0.05
Feng, 2019 [69]	ELISA	Cytokines	Oral squamous cell carcinoma (OSCC)	80 (60+, 20−)	70	86.7	AUC: 0.843; 95% CI: 0.743–0.942
Liu, 2019 [70]	ELISA	Heat shock protein 90 α(Hsp90α)	Pancreatic cancer		81.33	81.65	Cut-off: 69.19 ng/mL; AUC: 0.895
He, 2020 [71]	ELISA	TOPO48 (48 kDa-fragment derived from human DNA-topoisomerase I)	Breast cancer	214 (78+, 136−)	76	100	AUC: 0.801; 95% CI: 0.716–0.887
Selickaja, 2022 [72]	In-house ELISA	Anti-TIF1-γ autoantibodies	Dermatomyositis	213 (131+, 82−)	58	86	The anti-TIF1-γ results from the in-house ELISA were confirmed with IP in 93 of 101 (92%) cases, κ = 0.76, with a commercial ELISA in 110 of 131 (84%) cases, κ = 0.63
	Commercial ELISA				63	72	
Li, 2022 [73]	CRISPR-ELISA	Cytokines (IL-6, IL-8, and IL-10)	Lung cancer	252 (127+, 125−)	80.60	82	AUC: 0.79
Ye, 2023 [74]	ELISA	Fibronectin 1 (FN1)	Papillary thyroid cancer	49 (26+, 23−)	96.89	91.67	AUC: 0.924; 95% CI: 0.867–0.98
Vanarsa, 2023 [75]	ELISA	Urine proteins (e.g., IL-8, IgA, fibronectin)	Bladder cancer	68 (37, 31−)	97	90	AUC: 0.96; 95% CI: 0.92–1.00
Gerber, 2023 [76]	Sandwich ELISA	Plasma gelsolin	Epithelial ovarian cancer	96 (70+, 26−)	73.91	72.46	AUC: 0.7656, *p* = 0.0001, cut-off 1.586
Makoui, 2023 [77]	ELISA	Anti-Ki67 mAbs (2H1)	Breast cancer	200 (+/− not given)	94.20	99	Accuracy: 96.6%, *p* = 0.001, r = 0.298
		anti-P53 mAbs(2A6)			97.30	98.10	Accuracy: 97.5%
Liu, 2023 [78]	ELISA	AFP+IL-34	Hepatocellular carcinoma	108 (88+, 20−)	63.2	93.10	AUC: 0.837, cut-off value 1.88
Molga-Magusiak, 2023 [79]	ELISA	sPD-L1	Head and neck cancer	60 (40 malignant, 20 benign)	35.50	95	AUC: 0.664; 95% CI: 0.529–0.8; *p*-value = 0.039; cutoff: 0.765 ng/mL
Wei, 2023 [80]	ELISA	Urinary exosomal prostate-specific antigen (UE-PSA)	Prostate cancer	272 (87+, 185−)	95	-	UE-PSA could avoid 57.6% (155 cases) of unnecessary biopsies while only missing 2.6% (7 cases) of clinically significant prostate cancer (csPC)
Rezuchova, 2023 [81]	ELISA	Carbonic anhydrase IX (CA IX)	Breast cancer	100 (24+, 76−)	70	90	AUC: 0.822; 95% CI: 0.4601–0.6700; *p* < 0.001
Debernardi, 2023 [82]	ELISA	Urinary biomarkers-LYVE1, REG1B, TFF1; plasma biomarkers-CA19-9 PancRISK+CA19-9	Pancreatic ductal adenocarcinoma (PDAC)	198 (99+, 198−)	72	90	AUC: 0.827; 95% CI: 0.726–0.927
Tan, 2023 [83]	ELISA	Tumor necrosis factor receptor-associated protein 1 (TRAP1)	Limited-stage disease SCLC (small cell lung cancer)	367 (292+, 75−)	96.40	56	AUC: 0.819; cutoff 349.03 pg/mL
Xu, 2023 [84]	ELISA	7 tumor-associated autoantibodies (AABs)	lung cancer	987 (533+, 454−)	64	51.5	The positive predictive value (PPV) of 7-AABs was 63.97% and the negative predictive value (NPV) was 51.54%. The false positive rate (FPR) for 7-AABs was 36.02% and the false negative rate (FNR) was 48.49%
				**Average, median**	**77%, 76%**	**84%, 88%**	
				**Average AUC (# of studies)**	**0.83 (12)**		
**Immunoassays**							
Furuya, 2019 [85]	Multiplex bead-based IA(MBA)	Proteins	Bladder cancer	80 (40+, 40−)	93	95	Accuracy: 94%; AUC: 0.97
	Multiplex electro-chemiluminescent assay (MEA)				85	80	Accuracy 83%; AUC: 0.86
Tan, 2022 [86]	Electro-chemiluminescence IA	Antithyroglobulin (TGAb) and antithyroid peroxidase (TPOAb)	Hashimoto’s thyroiditis (HT)	1874 (806+, 1068−)	53.80	72.1	PPV 86.8%, NPV 31.4%, FPR 27.9%, FNR 46.2%
Jagodzińska, 2023 [87]	Fluorescent IA	FGF 21 (metabolism regulator)	Endometrial cancer	233 (186+, 47−)	41	90	AUC: 0.677
Tan, 2023 [83]	Chemiluminescence IA	Neuron-specific enolase (NSE)	LS SCLC	367 (292+, 75−)	60.70	92.40	AUC: 0.800; cutoff 5.93 ng/mL
		Carcinoembryonic antigen (CEA)			92.30	18.90	AUC: 0.513; cutoff 10.33 ng/mL
		Carbohydrate antigen 19-9 (CA19-9)			100	18.5	AUC: 0.535; Cutoff: 7.46 U/ml
		TRAP1+NSE			90.8	92.9	-
		Combination of 4 biomarkers			96.40	81.50	-
Mitobe, 2023 [88]	Chemiluminescence IA	sIL-2R	Central nervous system lymphoma	130 (48 PCNSL 8 SCNSL, 16 metastatic brain tumors 58 glioblastoma (GBM)	87.5	66.7	AUC: 0.826; cutoff 521 U/mL
Sparano, 2023 [89]	Chemiluminescence IA	Calcitonin	Thyroid carcinoma	55 (28 complete response, 27 persistent disease)	89	82	Early calcitonin level ≥ 16 pg/mL AUC = 0.911, CI95%: 0.819–1000, *p* < 0.001
Sekacheva, 2023 [90]	Chemiluminescent IA	CA-62	Breast cancer	269 (196+, 73−)	92	93	For comparison: Sensitivity for mammography was 63–80% at 60% specificity
Kim, 2023 [91]	3D-plus-3D IA	Engrailed-2 protein	Prostate cancer	90 (60+, 30−)	100	100	Incubation time 12–16 h
Xu, 2023 [84]	electro-chemiluminescence IA	7 tumor-associated autoantibodies (AABs) + 7 tumor antigens (7-TAs)	Lung cancer	987 (533+, 454−)	92.09	52.06	Specificity 95% CI 45.58–58.48%
				**Average, median**	**84%, 91%**	**74%, 82%**	
				**Average AUC (# of studies)**	**0.76 (5)**		

Abbreviations: CRISPR-ELISA—clustered regularly interspaced short palindromic repeats ELISA, AUC—area under the receiver operating curve (ROC), CI—confidence interval.

**Table 3:** The nucleic acid-based methods for the cancer diagnosis.

Author, Year	Technique	Analyte	Detection	Sensitivity	Specificity	Accuracy	Number of Samples
**Quantitative PCR**							
Alhasan, 2016 [99]	Real-time qPCR	Circular miRNas (miR-200c, miR-605, miR-135a, miR-433, and miR-106a)	High-risk prostate cancer from low-risk			89%	16 (8+; 8−)
Zhu, 2016 [100]	TaqMan probe qRT-PCR	Four miRNAs (miR-182, miR-183, miR-210, or miR-126) with carcinoembryonic antigen (CEA)	Non-small cell lung cancer	81.3%	100%	90.8%	216 (112+; 104−)
Liang, 2017 [101]	qPCR	*Fusobacterium nucleatum*	Colorectal Cancer	82.0%	80.7%	81.3%	230 (111+; 119−)
	qPCR	*Fusobacterium nucleatum*, *Bacteroides clarus*, *Roseburia intestinalis*, *Clostridium hathewayi*		83.8%	83.2%	83.5%	
	Quantitative PCR and HemoSure immunogold labeling FIT dipsticks	*Fusobacterium nucleatum, Bacteroides clarus, Roseburia intestinalis, Clostridium hathewayi*		92.8%	81.5%	87.0%	
Ooki, 2017 [102]	Quantitative methylation-specific PCR	6 genes: CDO1, HOXA9, AJAP1, PTGDR, UNCX, and MARCH11	non–small cell lung cancer (stage IA lung adenocarcinoma)	72.1%	71.4%	71.8%	85 (43+; 42−)
Wang, 2017 [103]	qRT-PCR	2 miRNAs (miR-19b-3p and miR-106a-5p)	Gastric cancer	95%	90%	92.5%	40 (20+; 20−)
Lithwick Yanai, 2017 [104]	RT-qPCR RosettaGX Reveal	miRNA hsa-miR-375	Thyroid cancer	97.5%	78.2%	83.3%	150 (39+; 111−)
Bartak, 2017 [105]	MethyLight PCR	SFRP1, SFRP2, SDC2, and PRIMA1	Colorectal Cancer	91.5%	97.3%	94.1%	84 (47+; 37−)
Agthoven, 2017 [106]	Real-time qPCR	Micro RNAs (miR-371a-3p, miR-373-3p, miR-367-3p)	Testicular germ cell cancer	90%	91%	90.3%	414 (250+; 164−)
Kahraman, 2018 [107]	RT-qPCR	77 miRNAs	Triple negative breast cancer	83.8%	74.2%	79%	
Xie, 2018 [108]	Quantitative real-time PCR	Two long non-coding RNA and three tumor markers (CEA, CYFRA21-1, and SCCA)	Non-small cell lung cancer	91%	70%	80.5%	200 (100+; 100−)
Pan, 2019 [109]	qRT-PCR	Exosomal circular RNA (hsa-circ-0004771)	Colorectal cancer	80.91%	82.86%	81.4%	145 (115+; 35−)
Zhang, 2019 [110]	Real-time qPCR	three long non-coding RNA panel (PCAT-1, UBC1 and SNHG16)	Bladder cancer	80%	75%	77.5%	320 (16+; 160−)
Liu, 2020 [111]	Methylation-specific PCR	Methylated COL4A2, TLX2	Colorectal cancer	91.7%	97.6%	94.5%	163 (80+; 83−)
Liao, 2020 [112]	RT-PCR	miRNAs (Integrated 2 sputum miRs-31-5p and 210-3p and one plasma miR-21-5p)	Non-small cell lung cancer	85.5%	91.7%	0.913	111 (56+; 55−)
Wang, 2020 [113]	qRT-PCR	PIWI-Interacting RNAs piR-020619 and piR-020450 in serum	Small colorectal tumors	81.7%	90.9%	0.869	71+
			Early-stage (I and II) colorectal cancer	76.8%	90.9%	0.839	173+
Xie, 2020 [114]	Methylation-specific qPCR	13 Methyl DNA markers	Colorectal cancer	90%	90%	90% 0.96	100 (40+; 60−)
Huang, 2020 [115]	RT-qPCR	4 miRNAs (miR-203a-3p, miR-145-5p, miR-375-3p and miR-200c-3p) in serum	Colorectal cancer	81.3%	73.3%	77.5%; 0.893	160 (80+; 80−)
Yao, 2021 [116]	Probe-based duplex PCR	Five bacteria (*Prevotella copri, Gemella morbillorum, Parvimonas micra, Cetobacterium somerae, and Pasteurella stomatis*) and fecal immunochemical test	Colorectal cancer	68.0%	89.3%	75.5%	318 (206+; 112−)
Peng, 2022 [117]	PCR	Methylated biomarkers (ELMO1, ZNF582 and TFPI2)	Upper gastrointestinal cancer	71.0%	90%	80.6%	376 (186+; 190−)
Miyoshi, 2022 [118]	qRT-PCR	8 miRNAs in serum (miR-103, miR-106b, miR-151, miR-17, miR-181a, miR-21, miR-25, and miR-93)	Esophageal squamous cell carcinoma (ESCC)	89.3%	84.3%	86.6%	186 (84+; 102−)
Ruiz-Banobre, 2022 [119]	Methylation-specific RT-PCR	Methylation of RNA LINC00473 (tissue)	Colorectal cancer	91%	100%	0.941	
Iadsee, 2023 [120]	Real-time qPCR	Fecal *Erysipelatoclostridium ramosum*	Colorectal cancer	72.7%	64.7%		
		Tissue *Erysipelatoclostridium ramosum*	Colorectal cancer	86.7%	65.5%		
**qPCR average**			Average/Median	83.8% 83.8% (25)	83.9% 83.2% (25)	83.7% 83.4% (20)	
**Droplet digital PCR**							
Uehiro, 2016 [121]	Digital PCR	Circulating free DNA (12 methylated cancer markers, 4 control markers)	Breast cancer	86.2%	82.7%	84.7%	111 (58+; 53−)
Sefrioui, 2017 [122]	Digital PCR	Circulating tumor DNA	Pancreatic adenocarcinoma	65.1%	75.0%	67.2%	55 (43+; 12−)
Kalinich, 2017 [123]	Digital PCR	Circulating tumor cells	Hepatocellular carcinoma	56%	95%	86.3%	73 (16+; 57−)
Sun, 2020 [124]	Droplet digital rt-PCR	Hepatocellular carcinoma (HCC)-specific extracellular vesicle (EV) mRNA markers	Hepatocellular carcinoma	94.4%	88.5%	91.9%	62 (36+; 26−)
Shinjo, 2020 [125]	Methyl-CpG binding protein with droplet digital PCR	5 DNA methylation markers in tissues	Pancreatic cancer	96.4%	90%	95.9%	147 (137+; 10-)
		5 DNA methylation markers in serum		68%	86%	78.7%	61 (47+; 14−)
Ruiz-Banobre, 2022 [119]	Droplet digital RT-PCR	Methylation of RNA LINC00473 (plasma)	Colorectal cancer	90%	63%	0.833 (AUC)	
**ddPCR average**			Average/Median	79.4% 86.2% (7)	82.9% 86.0% (7)	84.2% 85.5% (6)	
**All PCR**			Average/Median	82.9% 83.8% (32)	83.7% 83.8% (32)	83.9% 83.4% (26)	
**LAMP**							
Saito, 2021 [126]	LAMP	Epidermal growth factor receptor mutations exon 19 deletion or L858R point mutation	Non-small cell lung cancer	95.9%	97.1%	96.6%	117 (49+; 68−)
Sebuyoya, 2022 [127]	Capture of LAMP products on the surface of gold screen printed electrodes and electrochemical (amperometry) biosensor	DNA of human papillomavirus HPV16 and HPV18	Cervical cancer	91.7%	94.4%		15
Lin, 2022 [128]	RT-LAMP with machine learning	11 mRNA biomarkers	Lung cancer	88.0%	86.6%	AUC 0.934	86
Moranova, 2022 [129]	LAMP with chronoamperometry	Long non-coding RNA prostate cancer antigen 3	Prostate cancer	100%	100%	100%	19 (12+; 7−)
**LAMP average**			Average Median	93.9% 93.8% (4)	94.5% 95.8% (4)	98.3% 98.3% (2)	
**All clinical**			Average Median	84.1% 85.9% (36)	84.8% 86.3% (36)	84.9% 84.1% (28)	

Note: For some studies without the reported accuracy, AUC value is provided instead. AUC was not included in the calculation of average accuracy. The number in brackets that follows after the average diagnostic performance designates the number of clinical sensitivity, specificity, or accuracy values used for measuring the average.

**Table 5:** Clinical detection of cancer biomarkers using FTIR.

First Author, Year	Assay/Data Processing Method	Analyte (Cancer Type)	Cancer Patients/Control Patients	Sensitivity/Specificity/Accuracy
Smith, 2016 [166]	ATR-FTIR (diamond)	Protein, lipid, phosphate, carbohydrate, amide (Brain cancer)	433 (134 primary brain cancer, 177 metastatic brain cancer, 64 high-grade glioma, 23 low-grade glioma)	92.8%/91.5%/92.4%
Elmi, 2017 [167]	ATR-FTIR	Proteins, lipids, collagen, esters etc. (Breast cancer)	86 (43+, 43−)	76%/72%/80%
Liu, 2017 [168]	Transmission FTIR (barium fluoride)	Lipids, proteins, sugars, etc. (Gastric cancer)	70 (40+, 30−)	95%/70%/84.2%
Paraskevaidi, 2018 [169]	ATR-FTIR	Proteins and nucleic acids (Endometrial cancer, ovarian cancer)	30 (10 endometrial cancer, 10 ovarian cancer, 10 healthy)	95%/100%/95%-endometrial cancer, 100%/96.3%/100%-ovarian cancer
Adeeba, 2018 [170]	ATR-FTIR	DNA and RNA (Oral cancer)	147 (67 oral cancer, 60 “niswar” (dipping smokeless tobacco product) users, 20 control)	100%100%/95%
Butler, 2019 [171]	ATR-FTIR	Protein (Brain cancer)	724 (104+, 620−)	93.2%/92.8%/96%
Bangaoil, 2020 [172]	ATR-FTIR	Hematoxylin and eosin (H&E)-stained tissues (Lung cancer)	120 (54+, 66−)	97.73%/92.45%/94.85%
Sitnikova, 2020 [173]	ATR-FTIR	Several functional groups of DNA and RNA (Breast cancer)	146 (66+, 80−)	92.3%/87.1%/89.3%
Tomas, 2022 [174]	ATR-FTIR via NN	Lipid, nucleic acid, phospholipid, and carbohydrates (Breast cancer)	166 (88 malignant, 78 benign)	-/89%/96%
Sala, 2022 [175]	ATR-FTIR	Proteins, lipids (Pancreatic cancer)	235 (100 pancreatic cancer and 100 healthy)	92%/88%/90%
Du, 2022 [176]	FTIR	Lipids, proteins, sugar, and nucleic acids (Breast cancer)	526 (308 invasive breast cancer, 101 ductal carcinoma in situ, and 117 healthy controls)	100%/100%/94.2%
Chen, 2022 [177]	FTIR	Proteins, lipids, nucleic acids (Esophageal cancer)	68 (48-stage II, 20-stage III)	98.53%/100% /99.26%- late-stage cancer 91.43%/100%/97.08%-early-stage cancer
Guo, 2022 [178]	ATR-FTIR	Protein (Liver cancer—LC, gastric cancer—GC, colorectal cancer—CC)	252 (25 LC, 68 GC, 73 CC, 44 control)	100%/95%/95%
NMP de Souza, 2023 [179]	ATR-FTIR	Nucleic acid (Breast cancer)	74 (56+, 18−)	-/-/100%
Dong, 2023 [180]	FTIR	Protein, lipids, nucleic acids (Gastric cancer)	160 fresh non-metastatic and 80 metastatic lymph nodes, (each) from 60 patients with gastric cancer	96.6%/93.8%/-
		FTIR (15 reports)	Arithmetic mean (overall):	94.71%/91.75%/93.64%
			Median (overall):	95.00%/93.30%/95.00%

**Table 6:** Summary of the clinical performance of different detection techniques of cancer biomarkers.

Technique	Total Studies	Average Sensitivity (Median), %	Average Specificity (Median), %	Average Accuracy (Median), %	Average sens. + spec., %	Ave. AUC (# Publications)	All Refs (Refs. with Reported AUC)
Mass spectrometry	33 (7 with acc)	86 (87.0)	87 (87.5)	87 (87.3)	86.6	0.87 (28)	[9,30,31,32,33,34,35,36,37,38,39,40,41,42,43,44,45,46,47,48,49,50,51,52,53,54,55,56,57,58,59,60,61]
PCR	31 (25 with acc)	83 (83.8)	84 (83.8)	84 (83.4)	83.5	0.89 (21)	[99,100,101,102,103,104,105,106,107,108,109,110,111,112,113,114,115,116,117,118,119,120,121,122,123,124,125,126,127,128,129]
ELISA	17 (2 with acc)	77 (76.0)	84 (88.4)	97 (-)	80.5	0.83 (12)	[34,69,70,71,72,73,74,75,76,77,78,79,80,81,82,83,84]
IAs	9 (3 with acc)	84 (91.4)	74 (81.8)	77 (79.0)	79.0	0.76 (5)	[83,84,85,86,87,88,89,90,91]
SERS	19 (4 with acc)	91 (92.0)	93 (94.0)	97 (98.0)	91.5	0.97 (3)	[138,139,140,141,142,143,144,145,146,147,148,149,150,151,152,153,154,155,156]
FTIR	15 (14 with acc)	95 (95)	92 (93.3)	94 (95)	93.2	0.95 (3)	[166,167,168,169,170,171,172,173,174,175,176,177,178,179,180]

Abbreviations: PCR—polymerase chain reaction, ELISA—enzyme-linked immunosorbent assay, IAs—immunoassays, SERS—surface-enhanced Raman spectroscopy, FTIR—Fourier-transform infrared resonance spectroscopy, acc—accuracy (%).

**Table 7:** Summary of the research papers with detection using machine learning (ML).

Authors-Year	Analytical Technique	Analyte	Sensitivity	Specificity	Accuracy	Number of Samples	AUC
Banaei, 2019 [190]	SERS, K-nearest neighbor, and classification tree ML algorithms	5 protein biomarkers (CA19-9, HE4, MUC4, MMP7, and mesothelin)	86	93	91	20 sera samples	
Xie, 2021 [188]	LC-MS/MS, Naïve Bayes ML algorithm	Specific combination of six metabolic biomarkers	98.1	100		97	0.989
Ahamad, 2022 [191]	Random Forest (RF)	Carbohydrate antigen 125, carbohydrate antigen 19-9, carcinoembryonic antigen, and human epididymis protein 4	86		91	106 patients with OC marker features	0.93
Ahamad, 2022 [191]	Random Forest (RF), Gradient Boosting Machine (GBM), and Light Gradient Boosting Machine (LGBM)	Carbohydrate antigen 125, carbohydrate antigen 19-9, carcinoembryonic antigen, and human epididymis protein 4	97		88	349 patients with 49 features	0.87
Kim, 2022 [192]	Artificial neural networks (ANN), Random Forest (RF), and support vector machine (SVM)	CA125	87	98		269 serum samples	
Kiritani, 2021 [193]	PESI-MS, logistic regression	Specific monounsaturated fatty acid-bonded phospholipids	99	100	99.5	NP 80/103	0.9999
Dong, 2023 [194]	SERS and artificial intelligence for cancer screening (SERS-AICS)		95.87	95.4	95.81	Early Lung C NP 45/36; Early Colorectum C NP 42/32 Gastric C NP 39/36 Liver C NP 33/32	0.81 0.94 0.89 0.93 Av of 4: 0.893
Rahman, 2021 [195]	Support vector machine (SVM) model with radial basis function (RBF) kernel	BMI, Age, Glucose, MCP-1, Resistin, and Insulin.	95.1	94	93.9		0.989
		Average	93.0	96.7	93.2		0.945
		Median	95.5	96.7	92.5		0.960

C is cancer; NP x/y is number of patients; x N is patients with cancer; y N is patients without cancer (healthy control).

## Data Availability

No new data were created or analyzed in this study. Data sharing is not applicable to this article.
